# Comparison of the efficacy among multiple chemotherapeutic interventions combined with radiation therapy for patients with cervix cancer after surgery: A network meta-analysis

**DOI:** 10.18632/oncotarget.17259

**Published:** 2017-04-20

**Authors:** Lei Chang, Ruixia Guo

**Affiliations:** ^1^ Department of Gynecology, The First Affiliated Hospital of Zhengzhou University, Zhengzhou, Henan 450000, China

**Keywords:** cervix cancer, radiotherapy, chemotherapy, efficacy, network meta-analysis

## Abstract

**Background:**

Cervix cancer was the second most common cancer in female. However, there was no network meta-analysis (NMA) comparing the efficacy of the multiple chemotherapeutic interventions combined with radiation therapy in patients after operation.

**Methods:**

Randomized controlled trials were retrieved from PubMed, Embase and Cochrane Library. Overall survival (OS), recurrence-free survival (RFS), incidence of recurrence and distant metastasis were the main outcomes, particularly 5-year OS and PFS were considered as primary outcomes. Furthermore, the hazard ratio (HR) or odds ratio (OR) and their 95% credible intervals (CrIs) were extracted. The surface under cumulative ranking curve (SUCRA) was also used in this NMA.

**Results:**

A total of 39 eligible trials with 8,952 patients were included and 22 common chemotherapies were evaluated in this meta-analysis. For OS, cisplatin+fluorouracil+hydroxyurea, fluorouracil+mitomycin C, cisplatin and cisplatin+fluorouracil were better than placebo. As for RFS, cisplatin+fluorouracil, fluorouracil+mitomycin C, and cisplatin alone had the significant superiority compared with placebo. In terms of incidence of recurrence, the optimal drug combination was cisplatin+ifosfamide (0.93) based on SUCRA. Moreover, epirubicin (OR = 0.28, 95% CrI: 0.08-0.91) was the only one had the distinguished potency in reducing the occurrence of distant metastasis with a SUCRA rank probability of 0.88.

**Conclusion:**

We recommended cisplatin+fluorouracil+hydroxyurea and cisplatin+docetaxel for their good efficacy in long term survival. Meanwhile, the combination of multiple drugs with different mechanisms worked better.

## INTRODUCTION

Cervix cancer is caused by abnormal proliferation of cells with capacity of invasion, arising from the lower, narrow end of uterus to vagina. According to the definition of the National Cancer Institution of American National Institutes of Health, cervix cancer contains two main types, the squamous cell carcinoma originating from thin, flat cells that line the cervix and the adenocarcinoma arose from cells that make mucus and other fluids [1, 2]. And it is the second most frequent cancer occurred in female worldwide, next to breast cancer, accounting for 7.9% cancer cases and 7.5% cancer deaths in female in 2012 [3, 4]. With the development of medical and healthcare system, the average 5-year survival rate of cervical cancer has reached 66% in developed countries, yet less than half patients from developing countries could live longer than 5 years [5, 6]. The international biological study on etiology reported that human papillomavirus (HPV) was responsible for about 93% invasive cervical cancers [7]. Additionally, cigarette smoking, no matter active or passive, long-term use of oral contraceptives and multiple pregnancies are also high risk factors for cervix cancer [8, 9].

On the fundamental of clinical examination instead of the surgical findings, the International Federation of Gynecology and Obstetrics (FIGO) divides the cervix cancer into five stages from 0 stage to IV stage [10]. Surgery is a common treatment, while the specific operation and scope for different FIGO stages are distinctive. Except for conization and hysterectomy, one of the traditional surgeries usually performed on stage IA microinvasive cancer, there are many methods of fertility preservation for cervical cancer, as radical vaginal trachelectomy [11]. Besides, radiotherapy is also an available way to treat cervical cancer and preserve reproductive function, splitting into external and internal radiation therapy, which are given depending on both type and stage of the cancer [12].

For further improvement of survival length and decreasing the incidence of recurrence and distant metastasis, chemotherapy is often combined with radiotherapy after surgery through multiple mechanisms exerting the synergistic effect. Cisplatin, fluorouracil, hydroxyurea, bleomycin, ifosfamide and their combination with other drugs are common medical choices with different pharmacological reactions, and the combination of chemo and radiotherapy could be further categorized into neoadjuvant, adjuvant and concurrent therapy due to whether the medication was performed before, after or concurrent with the process of radiation [13–14].

Fortunately, regardless of the administration time point, a large number of randomized control trials (RCTs) assessing the efficacy and safety between different chemotherapeutic agents after radiotherapy or radiotherapy alone had been carried out and provided with sufficient clinical data. However, some of them were contradicted to each other. Two trials appraised the concurrent radiation and cisplatin plus fluorouracil compared with radiation alone. Morris et al. reported a significant superiority in both overall survival (OS) and recurrence-free survival (RFS), but the same outcomes of Kim et al.'s trial were 1.03 and 1.01 without statistically significance. Meanwhile, although many meta-analyses (MA) existed, there was still a lack of a conclusive treatment strategy for patients with cervical cancer, and none of them conducted a network meta-analysis (NMA) among variety of chemotherapies to draw the best outcomes result in patients [15, 16].

As the first NMA on this subject, we synthesized both direct and indirect data to compare the efficacy among multiple common chemotherapies combined with radiotherapy in patients with cervical cancer after surgery. OS, RFS, the incidence of recurrence, and distant metastasis were used as the outcomes to assess cisplatin, cisplatin+fluorouracil, hydroxyurea, fluorouracil alone, cisplatin+ifosfamide, bleomycin+ifosfamide+cisplatin, mitomycin C+fluorouracil, and other 14 chemotherapeutic strategies. 5-year OS and PFS were considered as primary outcomes. This multiple-treatment Bayesian meta-analysis aimed to integrate the existed data and to draw a conclusion to offer a guideline for the corresponding clinical practice.

## RESULTS

### Included studies

**Figure 1:**
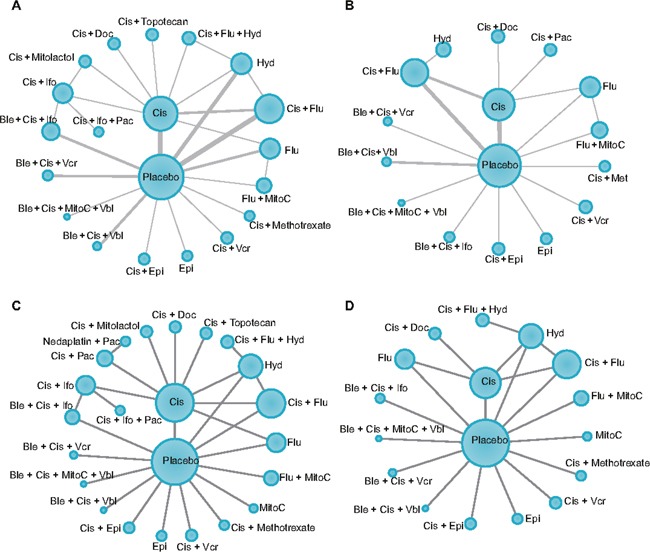
Network plots for each outcome **(A)** recurrence; **(B)** distant metastasis; **(C)** 1-year and 3-year overall survival (OS) and recurrence-free survival (RFS); **(D)** 5-year OS and RFS. Each circle represents a specific treatment, and the size of the circles depends on the sample size involved in a specific treatment. The width of the line depends on the number of included studies for each comparison. OS, overall survival; RFS, recurrence-free survival.

This NMA included 39 eligible trials with 8,952 patients involved, screened from the 412 identified literatures from electronic database depending on the inclusion and exclusion criteria, which were published between March 1979 and October 2015 were included [13, 14, 17–53], and the efficacy of a total of 22 chemotherapies were evaluated. Besides, 113 meta-analysis or systematic reviews were retrieved through the keywords searching on the Internet. The characteristics of patients, the details of the specific interventions and the analyzed outcomes of each trial were listed in Table 1. Among 39 eligible trials, 34 of them gave the data of OS, 36 trials showed the PFS, 34 pairs of comparisons involved the data of recurrence, and the outcome of distant metastasis was analyzed in 24 trials. The directly compared connections among each chemotherapeutic agent for each outcome were displayed in Figure 1.

**Table 1:** Baseline characteristics of included studies

Study				Patient			Intervention				Outcome			
Study	Country	RCT	Follow-up, m	N	FIGO stage	Mean age, y	RT	Type	Contrast	Dosage, mg/m^2^ or else	OS	RFS	Recurrence	Distant metastasis
Pu 2013	China	√	60	140, 145	IB-IIA	45, 47	√	c	Cis vs Cis + Doc	40, 30 + 30	√	√	√	√
Yin 2012	China	-	120	148, 104	IB2-IIB	NA	-	n	Cis + Pac vs Nedaplatin + Pac	50 + 175, 50 + 175	√	√		
Symonds 2000	UK	√	100	104, 100	IIB-IVA	49, 48	√	n, r	Cis + Methotrexate vs Placebo	50 + 100, -	√	√	√	√
Roberts 2000	USA	√	46	78, 82	IB2-IVA	47.5, 48.5	√	a, r	MitoC vs Placebo	15, -	√	√		
Morris 1999	USA	√	60	195, 193	IB-IVA	47, 47	√	c, r	Cis + Flu vs Placebo	75 + 4000, -	√	√	√	
Tseng 1997	China	√	60	60, 62	IIB/IIIB	NA	√	c, r	Ble + Cis + Vcr vs Placebo	25 + 50 + 1, -	√	√	√	√
Thomas 1998	Canada	√	80	50, 49	IB-IVA	NA	√	c, r	Flu vs Placebo	1000, -		√	√	
Sundfor 1996	Norway	√	60	47, 47	IIIB/IVA	52.7, 52.5	√	n, r	Cis + Flu vs Placebo	100 + 1000, -	√	√	√	√
Rose 1999	USA	√	48	176, 173, 177	IIB-IVA	NA	√	c	Cis vs Cis + Flu + Hyd vs Hyd	40, 50 + 4000+ 2000, 3000	√	√	√	
Keys 1999	USA	-	48	183, 186	IB	NA	√	c, r	Cis vs Placebo	40, -	√	√	√	√
Long 2005	USA	√	36	147, 146	IVB	46, 48	√	c	Cis + Topotecan vs Cis	50 + 0.75, 50	√	√	√	
Kim 2008	Korea	√	72	78, 77	IIB-IVA	58, 57	√	c	Cis + Flu vs Cis	20 + 1000, 30	√	√	√	√
Lanciano 2005	USA	√	48	159, 157	IIB-IVA	NA	√	c	Cis vs Flu	40, 225	√	√	√	√
Peters 2000	USA	√	96	127, 116	IA2-IIA	41, 38	√	c, r	Cis + Flu vs Placebo	70 + 1000, -	√	√	√	√
Whitney 1999	USA	√	72	177, 191	IIB-IVA	NA	√	a	Cis + Flu vs Hyd	50 + 4000, 3000	√	√	√	√
Eifel 2004	USA	√	96	195, 195	IIB-IVA	NA	√	c, r	Cis + Flu vs Placebo	75 + 4000, -	√	√	√	√
Eddy 2007	USA	√	72	145, 143	IB	NA	√	n, r	Cis + Vcr vs Placebo	50 + 1, -	√	√	√	√
Herod 2000	UK	√	84	89, 88	IB-IVA	47, 46	√	n, r	Ble + Cis + Ifo vs Placebo	30 + 50 + 5000, -	√		√	
Lorvidhaya 2003	Thailand	√	120	233, 221, 242	IIB-IVA	48, 49, 50	√	c, a, r	Flu + MitoC vs Flu vs Placebo	300 mg/d + 10, 200 mg/d, -	√	√	√	√
Buda 2005	Italy	√	60	96, 108	IB2-IVA	47, 49	-	n	Cis + Ifo + Pac vs Cis + Ifo	75 + 5000+ 175, 75 + 5000	√	√	√	*
Leborgne 1997	Uruguay	√	60	48, 49	IB-IVA	47, 43	√	a, r	Ble + Cis + Vcr vs Placebo	25 + 50 + 1, -		√	√	
Nedovic 2012	Serbia	-	42	64, 70	IIB-IVA	51, 54	√	c	Cis + Flu vs Cis	75 + 4000, 40	√	√	√	√
Garipagaoglu 2004	Turkey	√	60	22, 22	IIB/IIIB	50.5, 49.2	√	c, r	Cis vs Placebo	20, -	√	√	√	√
Moore 2004	USA	-	24	130, 134	IVB	48.5, 46	-	n	Cis + Pac vs Cis	50 + 135, 50	√	√		√
Omura 1997	USA	√	24	151, 147, 140	IVB	46.3, 48.8, 47.3	-	n	Cis + Ifo vs Cis + Mitolactol vs Cis	50 + 5000, 50 + 180, 50	√	√	√	
Pearcey 2002	Canada	√	120	127, 126	IIB-IVA	NA	√	c, r	Cis vs Placebo	40, -	√	√	√	√
Bloss 2002	USA	√	24	141, 146	IVB	46, 45	-	n	Ble + Cis + Ifo vs Cis + Ifo	30 units + 50 + 5000, 50 + 5000	√	√	√	
Tattersall 1992	Australia	√	75	34, 37	IIB-IVA	54, 56	√	c, r	Ble + Cis + Vbl vs Placebo	15 + 50 + 4, -	√	√	√	√
Tattersall 1991	Australia	√	72	34, 37	IB-IIA	NA	√	a, r	Ble + Cis + Vbl vs Placebo	15 + 50 + 4, -		√	√	√
Tattersall 1995	Australia	√	48	129, 131	IIB-IVA	47, 52	√	c, r	Cis + Epi vs Placebo	60 + 110, -	√	√	√	√
Wong 1988	China	√	70	22, 25	IIB-IIIB	NA	√	c, r	Cis vs Placebo	25, -		√	√	√
Wong 1999	China	√	140	110, 110	I-IIIB	52.4, 55.5	√	a, r	Epi vs Placebo	90, -	√	√	√	√
Tabata 2003	Japan	√	60	32, 29	IIIB/IVA	57, 59	√	a, r	Ble + Cis + MitoC + Vbl vs Placebo	3 + 10+ 7 + 0.7, -	√		√	√
Piver 1987	USA	√	156	20, 25	IIIB	NA	√	c, r	Hyd vs Placebo	80 mg/kg, -	√	√	√	
Piver 1983	USA	√	110	20, 20	IIB	45.7, 50.5	√	c, r	Hyd vs Placebo	80 mg/kg, -	√		√	
Nagai 2001	Japan	√	120	32, 28	II-IV	55.6, 64.4	√	a	Cis vs Flu	120 mg, 200 mg/d	√	√		
Kumar 1994	India	√	24	94, 90	IIB-IVA	45, 45.5	√	n, r	Ble + Cis + Ifo vs Placebo	15 + 50 + 1000, -		√	√	√
Hreshchyshyn 1979	USA	-	48	51, 46	IIB-IVA	NA	√	c, r	Hyd vs Placebo	80 mg/kg, -	√	√	√	
Donnelly 2015	USA	-	348	42, 95, 99	IB1-IVA	49 (23-83)	√	c, c, r	Cis + Flu vs Cis vs Placebo	70 + 1000, 40, -	√	√		

Note: FIGO, International Federation of Gynecology and Obstetrics; RT, radiationtherapy; OS, overall survival; RFS, recurrence-free survival; a, adjuvant; n, neoadjuvant; c, concurrent; r, radiotherapy only.

*Data excluded to achieve network connectivity.

### Overall survival (OS)

OS were treated as the primary outcomes. Since 1-year OS was a short-term evaluation index showing no significant difference in most cases (Table 2), 3-year and 5-year OS were more reliable to reflect their efficacies. The hazard ratio (HR) of placebo was compared with 22 chemotherapies reported the HR of 3-year OS. As shown in the lower panel of Table 3, cisplatin+ifosfamide+paclitaxel (HR = 2.14, 95% CrI: 1.1-4.17), cisplatin+fluorouracil+hydroxyurea (HR = 1.78, 95% CrI: 1.08- 2.93), cisplatin+paclitaxel (HR = 1.77, 95% CrI: 1.23-2.54), fluorouracil+mitomycin C (HR = 1.75, 95% CrI: 1.04-2.95), cisplatin+ifosfamide (HR = 1.71, 95% CrI: 1.26-2.33), cisplatin+mitolactol (HR = 1.57, 95% CrI: 1.11-2.23), cisplatin+topotecan (HR = 0.69, 95% CrI: 0.50-0.96), cisplatin (HR = 0.70, 95% CrI: 0.56-0.87), and cisplatin+fluorouracil (HR = 1.32, 95% CrI: 1.07-1.64), these nine interventions with 95% CrI excluded 1.00 had dramatic advantage on improving the survival length of patients. Furthermore, for 5-year OS in the lower panel of Table 4, cisplatin+docetaxel (HR = 2.22, 95% CrI: 1.27-3.87), cisplatin+fluorouracil+hydroxyurea (HR = 1.81, 95% CrI: 1.15-2.86), fluorouracil+mitomycin C (HR =1.67, 95% CrI: 1.02-2.71), cisplatin (HR = 1.44, 95% CrI: 1.15-1.8), and cisplatin+fluorouracil (HR = 1.29, 95% CrI: 1.05-1.59) had statistically significance compared with placebo. Results of ranking analysis in Table 5 suggested that cisplatin+fluorouracil+hydroxyurea was efficacious in 1-year and 5-year period. Another two favorable interventions in 5-year OS were cisplatin+docetaxel and fluorouracil+mitomycin C, suggested a good therapeutic effect in long-term survival.

**Table 2:** Mixed evidence of 1-year overall survival and recurrence-free survival for different treatments

		1-year RFS																					
		Ble + Cis + Ifo	Ble + Cis + MitoC + Vbl	Ble + Cis + Vbl	Ble + Cis + Vcr	Cis	Cis + Doc	Cis + Epi	Cis + Flu	Cis + Flu + Hyd	Cis + Ifo	Cis + Ifo + Pac	Cis + Metho- trexate	Cis + Mito- lactol	Cis + Pac	Cis + Topo- tecan	Cis + Vcr	Epi	Flu	Flu + MitoC	Hyd	MitoC	Placebo
**1-year OS**	**Ble + Cis + Ifo**	**Ble + Cis + Ifo**		1.11 (0.46, 2.69)	1.53 (0.61, 3.85)	1.09 (0.6, 1.96)	1.6 (0.34, 7.59)	1.92 (0.66, 5.53)	0.96 (0.5, 1.86)	0.98 (0.28, 3.36)	0.97 (0.59, 1.57)	1 (0.35, 2.87)	1.32 (0.54, 3.19)	1.23 (0.57, 2.64)	1.12 (0.49, 2.57)	1.21 (0.55, 2.62)	1.59 (0.56, 4.52)	1.09 (0.46, 2.54)	1.36 (0.64, 2.89)	1.2 (0.4, 3.63)	1.09 (0.51, 2.29)	0.97 (0.37, 2.54)	1.43 (0.78, 2.62)
	**Ble + Cis + MitoC + Vbl**	0.67 (0.2, 2.22)	**Ble + Cis + MitoC + Vbl**																				
	**Ble + Cis + Vbl**	0.88 (0.26, 2.95)	1.31 (0.34, 4.95)	**Ble + Cis + Vbl**	1.38 (0.53, 3.56)	0.98 (0.48, 2)	1.44 (0.29, 7.19)	1.72 (0.58, 5.1)	0.86 (0.42, 1.77)	0.88 (0.25, 3.13)	0.87 (0.38, 2)	0.9 (0.25, 3.15)	1.18 (0.47, 2.96)	1.1 (0.46, 2.63)	1.01 (0.4, 2.54)	1.08 (0.45, 2.61)	1.43 (0.49, 4.17)	0.98 (0.4, 2.36)	1.23 (0.54, 2.79)	1.08 (0.35, 3.35)	0.98 (0.44, 2.18)	0.87 (0.33, 2.35)	1.29 (0.67, 2.46)
	**Ble + Cis + Vcr**	1.26 (0.26, 6.13)	1.87 (0.35, 10.02)	1.43 (0.27, 7.75)	**Ble + Cis + Vcr**	0.71 (0.33, 1.52)	1.04 (0.2, 5.33)	1.25 (0.41, 3.82)	0.63 (0.29, 1.34)	0.64 (0.17, 2.33)	0.63 (0.26, 1.51)	0.65 (0.18, 2.34)	0.86 (0.33, 2.22)	0.8 (0.32, 1.98)	0.73 (0.28, 1.91)	0.79 (0.32, 1.96)	1.04 (0.34, 3.12)	0.71 (0.28, 1.78)	0.89 (0.38, 2.11)	0.78 (0.25, 2.5)	0.71 (0.31, 1.65)	0.63 (0.23, 1.76)	0.93 (0.47, 1.87)
	**Cis**	0.88 (0.41, 1.88)	1.3 (0.48, 3.53)	1 (0.36, 2.75)	0.7 (0.17, 2.92)	**Cis**	1.47 (0.35, 6.22)	1.76 (0.7, 4.45)	0.88 (0.6, 1.3)	0.9 (0.3, 2.73)	0.89 (0.56, 1.42)	0.92 (0.32, 2.62)	1.21 (0.59, 2.48)	1.13 (0.7, 1.84)	1.03 (0.57, 1.85)	1.11 (0.67, 1.84)	1.46 (0.59, 3.62)	1 (0.51, 1.96)	1.26 (0.76, 2.08)	1.11 (0.42, 2.94)	1 (0.6, 1.67)	0.9 (0.4, 2.01)	1.32 (0.97, 1.79)
	**Cis + Doc**	1.02 (0.18, 5.72)	1.51 (0.24, 9.52)	1.16 (0.18, 7.35)	0.81 (0.1, 6.66)	1.16 (0.25, 5.45)	**Cis + Doc**	1.2 (0.22, 6.65)	0.6 (0.14, 2.67)	0.61 (0.1, 3.77)	0.61 (0.13, 2.76)	0.62 (0.1, 3.71)	0.82 (0.16, 4.12)	0.77 (0.17, 3.52)	0.7 (0.15, 3.32)	0.75 (0.16, 3.48)	0.99 (0.18, 5.46)	0.68 (0.14, 3.34)	0.85 (0.19, 3.93)	0.75 (0.13, 4.29)	0.68 (0.15, 3.14)	0.61 (0.12, 3.18)	0.9 (0.2, 3.91)
	**Cis + Epi**	0.83 (0.26, 2.58)	1.23 (0.34, 4.36)	0.94 (0.26, 3.39)	0.65 (0.13, 3.36)	0.94 (0.37, 2.38)	0.81 (0.13, 4.91)	**Cis + Epi**	0.5 (0.2, 1.26)	0.51 (0.13, 2.06)	0.5 (0.18, 1.4)	0.52 (0.13, 2.08)	0.69 (0.23, 2.03)	0.64 (0.23, 1.82)	0.58 (0.2, 1.74)	0.63 (0.22, 1.8)	0.83 (0.24, 2.8)	0.57 (0.2, 1.63)	0.71 (0.26, 1.95)	0.63 (0.18, 2.24)	0.57 (0.21, 1.53)	0.51 (0.16, 1.6)	0.75 (0.31, 1.78)
	**Cis + Flu**	0.93 (0.41, 2.11)	1.39 (0.51, 3.78)	1.06 (0.38, 2.95)	0.74 (0.18, 3.12)	1.06 (0.69, 1.64)	0.92 (0.18, 4.55)	1.13 (0.44, 2.89)	**Cis + Flu**	1.02 (0.34, 3.09)	1.01 (0.56, 1.8)	1.04 (0.34, 3.14)	1.37 (0.67, 2.81)	1.28 (0.69, 2.38)	1.17 (0.58, 2.35)	1.26 (0.67, 2.37)	1.65 (0.67, 4.1)	1.13 (0.58, 2.22)	1.42 (0.8, 2.52)	1.25 (0.47, 3.33)	1.13 (0.68, 1.89)	1.01 (0.45, 2.27)	**1.49 (1.1, 2.03)**
	**Cis + Flu + Hyd**	0.69 (0.19, 2.51)	1.02 (0.25, 4.22)	0.78 (0.19, 3.27)	0.54 (0.09, 3.15)	0.78 (0.26, 2.35)	0.67 (0.1, 4.48)	0.83 (0.21, 3.29)	0.74 (0.24, 2.22)	**Cis + Flu + Hyd**	0.99 (0.3, 3.26)	1.02 (0.22, 4.66)	1.35 (0.38, 4.79)	1.26 (0.37, 4.22)	1.15 (0.33, 4.02)	1.23 (0.36, 4.18)	1.62 (0.41, 6.49)	1.11 (0.32, 3.86)	1.4 (0.42, 4.6)	1.23 (0.29, 5.15)	1.11 (0.42, 2.97)	1 (0.27, 3.73)	1.46 (0.49, 4.36)
	**Cis + Ifo**	0.79 (0.4, 1.58)	1.17 (0.36, 3.84)	0.9 (0.27, 2.98)	0.63 (0.13, 3.02)	0.9 (0.45, 1.81)	0.77 (0.14, 4.22)	0.96 (0.31, 2.96)	0.85 (0.38, 1.86)	1.15 (0.32, 4.15)	**Cis + Ifo**	1.03 (0.4, 2.64)	1.36 (0.59, 3.13)	1.27 (0.65, 2.49)	1.16 (0.55, 2.45)	1.25 (0.63, 2.48)	1.64 (0.6, 4.48)	1.12 (0.51, 2.5)	1.41 (0.72, 2.77)	1.24 (0.43, 3.61)	1.12 (0.57, 2.21)	1.01 (0.4, 2.51)	1.48 (0.87, 2.51)
	**Cis + Ifo + Pac**	0.8 (0.25, 2.53)	1.18 (0.26, 5.32)	0.91 (0.2, 4.12)	0.63 (0.1, 3.91)	0.91 (0.29, 2.89)	0.78 (0.11, 5.39)	0.97 (0.22, 4.16)	0.85 (0.25, 2.88)	1.16 (0.24, 5.64)	1.01 (0.4, 2.54)	**Cis + Ifo + Pac**	1.32 (0.38, 4.64)	1.23 (0.39, 3.92)	1.12 (0.34, 3.74)	1.21 (0.38, 3.88)	1.59 (0.4, 6.3)	1.09 (0.32, 3.74)	1.37 (0.43, 4.36)	1.21 (0.29, 5)	1.09 (0.34, 3.47)	0.98 (0.26, 3.62)	1.44 (0.49, 4.22)
	**Cis + Metho- trexate**	1 (0.35, 2.85)	1.48 (0.45, 4.86)	1.14 (0.34, 3.78)	0.79 (0.16, 3.82)	1.14 (0.51, 2.57)	0.98 (0.17, 5.61)	1.21 (0.39, 3.75)	1.07 (0.47, 2.44)	1.46 (0.4, 5.33)	1.27 (0.45, 3.57)	1.25 (0.31, 5.02)	**Cis + Metho- trexate**	0.93 (0.39, 2.22)	0.85 (0.34, 2.15)	0.92 (0.38, 2.2)	1.21 (0.41, 3.52)	0.83 (0.34, 1.99)	1.04 (0.46, 2.36)	0.91 (0.29, 2.83)	0.83 (0.37, 1.84)	0.74 (0.28, 1.98)	1.09 (0.57, 2.08)
	**Cis + Mito- lactol**	1.01 (0.34, 3.03)	1.5 (0.42, 5.34)	1.15 (0.32, 4.14)	0.8 (0.16, 4.11)	1.15 (0.52, 2.53)	0.99 (0.17, 5.6)	1.22 (0.36, 4.14)	1.08 (0.44, 2.66)	1.47 (0.38, 5.69)	1.28 (0.44, 3.67)	1.27 (0.31, 5.14)	1.01 (0.33, 3.13)	**Cis + Mito- lactol**	0.91 (0.43, 1.95)	0.98 (0.49, 1.98)	1.29 (0.46, 3.62)	0.89 (0.39, 2.03)	1.11 (0.55, 2.24)	0.98 (0.33, 2.92)	0.88 (0.44, 1.8)	0.79 (0.31, 2.03)	1.17 (0.66, 2.07)
	**Cis + Pac**	1.05 (0.44, 2.55)	1.56 (0.52, 4.66)	1.2 (0.39, 3.63)	0.83 (0.19, 3.75)	1.2 (0.77, 1.88)	1.03 (0.21, 5.16)	1.28 (0.45, 3.58)	1.13 (0.6, 2.11)	1.53 (0.47, 5.04)	1.33 (0.58, 3.06)	1.32 (0.38, 4.57)	1.05 (0.42, 2.67)	1.04 (0.42, 2.59)	**Cis + Pac**	1.08 (0.5, 2.34)	1.42 (0.48, 4.18)	0.97 (0.4, 2.37)	1.22 (0.56, 2.64)	1.07 (0.34, 3.36)	0.97 (0.44, 2.12)	0.87 (0.32, 2.36)	1.28 (0.66, 2.48)
	**Cis + Topo- tecan**	1.03 (0.36, 2.93)	1.52 (0.45, 5.2)	1.17 (0.34, 4.04)	0.81 (0.16, 4.05)	1.17 (0.57, 2.4)	1.01 (0.18, 5.52)	1.24 (0.38, 4.02)	1.1 (0.48, 2.54)	1.5 (0.4, 5.56)	1.3 (0.48, 3.54)	1.29 (0.33, 5.02)	1.03 (0.35, 3.04)	1.02 (0.35, 2.95)	0.97 (0.42, 2.27)	**Cis + Topo- tecan**	1.32 (0.47, 3.72)	0.9 (0.39, 2.09)	1.13 (0.55, 2.31)	1 (0.33, 2.99)	0.9 (0.44, 1.85)	0.81 (0.31, 2.09)	1.19 (0.66, 2.14)
	**Cis + Vcr**	0.95 (0.27, 3.27)	1.4 (0.36, 5.47)	1.07 (0.27, 4.24)	0.75 (0.14, 4.13)	1.08 (0.38, 3.08)	0.93 (0.14, 6)	1.14 (0.31, 4.25)	1.01 (0.35, 2.91)	1.38 (0.32, 5.92)	1.2 (0.35, 4.1)	1.18 (0.25, 5.52)	0.95 (0.28, 3.24)	0.94 (0.25, 3.48)	0.9 (0.29, 2.81)	0.92 (0.26, 3.28)	**Cis + Vcr**	0.68 (0.24, 1.94)	0.86 (0.32, 2.32)	0.76 (0.21, 2.67)	0.68 (0.26, 1.81)	0.61 (0.2, 1.9)	0.9 (0.38, 2.11)
	**Epi**	0.87 (0.21, 3.68)	1.29 (0.27, 6.07)	0.99 (0.21, 4.7)	0.69 (0.11, 4.43)	0.99 (0.27, 3.57)	0.85 (0.11, 6.34)	1.05 (0.23, 4.74)	0.93 (0.26, 3.38)	1.27 (0.25, 6.49)	1.1 (0.26, 4.63)	1.09 (0.2, 6)	0.87 (0.21, 3.66)	0.86 (0.19, 3.89)	0.83 (0.21, 3.22)	0.85 (0.19, 3.68)	0.92 (0.19, 4.48)	**Epi**	1.26 (0.57, 2.75)	1.11 (0.37, 3.33)	1 (0.47, 2.14)	0.89 (0.34, 2.33)	1.32 (0.72, 2.39)
	**Flu**	1.18 (0.47, 2.97)	1.75 (0.58, 5.3)	1.34 (0.44, 4.13)	0.94 (0.21, 4.25)	1.35 (0.77, 2.36)	1.16 (0.22, 5.99)	1.43 (0.5, 4.08)	1.26 (0.65, 2.47)	1.72 (0.51, 5.77)	1.49 (0.62, 3.62)	1.48 (0.41, 5.31)	1.18 (0.46, 3.04)	1.17 (0.44, 3.08)	1.12 (0.55, 2.31)	1.15 (0.46, 2.86)	1.25 (0.39, 3.97)	1.36 (0.34, 5.35)	**Flu**	0.88 (0.31, 2.53)	0.8 (0.41, 1.56)	0.71 (0.29, 1.75)	1.05 (0.63, 1.74)
	**Flu + MitoC**	0.98 (0.29, 3.31)	1.45 (0.38, 5.55)	1.11 (0.29, 4.31)	0.77 (0.14, 4.21)	1.11 (0.4, 3.11)	0.96 (0.15, 6.12)	1.18 (0.33, 4.31)	1.05 (0.37, 2.94)	1.42 (0.34, 6.02)	1.24 (0.37, 4.16)	1.23 (0.27, 5.62)	0.98 (0.29, 3.29)	0.97 (0.27, 3.53)	0.93 (0.3, 2.85)	0.95 (0.27, 3.33)	1.03 (0.26, 4.12)	1.12 (0.23, 5.39)	0.83 (0.27, 2.57)	**Flu + MitoC**	0.9 (0.32, 2.56)	0.81 (0.25, 2.66)	1.19 (0.47, 3.01)
	**Hyd**	0.76 (0.33, 1.78)	1.13 (0.41, 3.16)	0.87 (0.31, 2.46)	0.6 (0.14, 2.59)	0.87 (0.53, 1.43)	0.75 (0.15, 3.78)	0.92 (0.35, 2.41)	0.82 (0.49, 1.35)	1.11 (0.42, 2.97)	0.97 (0.42, 2.2)	0.96 (0.28, 3.29)	0.76 (0.33, 1.78)	0.76 (0.3, 1.92)	0.72 (0.37, 1.41)	0.74 (0.31, 1.77)	0.81 (0.27, 2.37)	0.88 (0.24, 3.24)	0.65 (0.32, 1.31)	0.78 (0.27, 2.24)	**Hyd**	0.9 (0.37, 2.17)	1.32 (0.82, 2.12)
	**MitoC**	0.84 (0.26, 2.68)	1.24 (0.34, 4.52)	0.95 (0.26, 3.51)	0.66 (0.13, 3.46)	0.95 (0.37, 2.49)	0.82 (0.13, 5.05)	1.01 (0.29, 3.5)	0.9 (0.34, 2.36)	1.22 (0.3, 4.91)	1.06 (0.33, 3.36)	1.05 (0.24, 4.6)	0.84 (0.26, 2.66)	0.83 (0.24, 2.87)	0.79 (0.28, 2.29)	0.81 (0.25, 2.7)	0.89 (0.23, 3.36)	0.96 (0.21, 4.42)	0.71 (0.24, 2.07)	0.86 (0.23, 3.19)	1.1 (0.41, 2.95)	**MitoC**	1.47 (0.7, 3.1)
	**Placebo**	1.09 (0.52, 2.29)	1.61 (0.63, 4.1)	1.23 (0.48, 3.2)	0.86 (0.21, 3.46)	1.24 (0.87, 1.76)	1.06 (0.22, 5.19)	1.32 (0.56, 3.11)	1.16 (0.8, 1.68)	1.58 (0.54, 4.62)	1.38 (0.66, 2.86)	1.36 (0.42, 4.42)	1.09 (0.52, 2.26)	1.08 (0.45, 2.55)	1.03 (0.58, 1.82)	1.06 (0.48, 2.35)	1.15 (0.43, 3.09)	1.25 (0.36, 4.3)	0.92 (0.51, 1.67)	1.11 (0.42, 2.91)	1.42 (0.93, 2.18)	1.3 (0.53, 3.17)	**Placebo**

**Table 3:** Mixed evidence of 3-year overall survival and recurrence-free survival for different treatments

		3-year RFS																						
		Ble + Cis + Ifo	Ble + Cis + MitoC + Vbl	Ble + Cis + Vbl	Ble + Cis + Vcr	Cis	Cis + Doc	Cis + Epi	Cis + Flu	Cis + Flu + Hyd	Cis + Ifo	Cis + Ifo + Pac	Cis + Metho- trexate	Cis + Mito- lactol	Cis + Pac	Cis + Topo- tecan	Cis + Vcr	Epi	Flu	Flu + MitoC	Hyd	MitoC	Nedaplatin + Pac	Placebo
**3-year OS**	**Ble + Cis + Ifo**	**Ble + Cis + Ifo**		1.57 (0.78, 3.18)	1.66 (0.9, 3.05)	**1.47 (1.04, 2.09)**	1.29 (0.47, 3.55)	**2.39 (1.28, 4.47)**	1.32 (0.88, 1.98)	1.17 (0.63, 2.19)	1.04 (0.8, 1.34)	0.98 (0.54, 1.81)	1.57 (0.88, 2.78)	**2.24 (1.47, 3.4)**	1.16 (0.71, 1.92)	1.19 (0.74, 1.92)	1.89 (0.98, 3.65)	1.38 (0.66, 2.88)	**1.67 (1.08, 2.6)**	1.26 (0.74, 2.16)	**1.77 (1.14, 2.77)**	0.96 (0.47, 1.93)	3.06 (0.31, 30.58)	**1.91 (1.31, 2.8)**
	**Ble + Cis + MitoC + Vbl**	0.77 (0.31, 1.91)	**Ble + Cis + MitoC + Vbl**																					
	**Ble + Cis + Vbl**	1.3 (0.54, 3.13)	1.7 (0.53, 5.45)	**Ble + Cis + Vbl**	1.05 (0.49, 2.26)	0.94 (0.5, 1.76)	0.82 (0.26, 2.57)	1.52 (0.7, 3.29)	0.84 (0.45, 1.57)	0.75 (0.34, 1.65)	0.66 (0.34, 1.3)	0.63 (0.26, 1.5)	1 (0.48, 2.07)	1.42 (0.73, 2.78)	0.74 (0.36, 1.52)	0.76 (0.37, 1.54)	1.2 (0.54, 2.68)	0.88 (0.37, 2.09)	1.06 (0.56, 2.04)	0.8 (0.4, 1.62)	1.13 (0.58, 2.18)	0.61 (0.26, 1.4)	1.95 (0.18, 20.63)	1.22 (0.67, 2.2)
	**Ble + Cis + Vcr**	2.99 (0.78, 11.5)	3.9 (0.83, 18.39)	2.3 (0.5, 10.6)	**Ble + Cis + Vcr**	0.89 (0.53, 1.5)	0.78 (0.26, 2.3)	1.44 (0.72, 2.87)	0.8 (0.47, 1.34)	0.71 (0.35, 1.44)	0.62 (0.35, 1.11)	0.59 (0.27, 1.32)	0.95 (0.5, 1.8)	1.35 (0.76, 2.39)	0.7 (0.37, 1.32)	0.72 (0.39, 1.33)	1.14 (0.56, 2.34)	0.83 (0.38, 1.84)	1.01 (0.58, 1.75)	0.76 (0.41, 1.4)	1.07 (0.61, 1.87)	0.58 (0.27, 1.23)	1.85 (0.18, 19.05)	1.15 (0.72, 1.86)
	**Cis**	0.85 (0.61, 1.18)	1.11 (0.46, 2.65)	0.65 (0.28, 1.5)	0.28 (0.08, 1.06)	**Cis**	0.88 (0.34, 2.27)	1.62 (0.95, 2.78)	0.9 (0.71, 1.13)	0.8 (0.47, 1.35)	**0.7 (0.54, 0.92)**	0.67 (0.36, 1.23)	1.07 (0.66, 1.71)	1.52 (1.21, 1.91)	0.79 (0.55, 1.13)	0.81 (0.59, 1.12)	1.29 (0.72, 2.28)	0.94 (0.48, 1.82)	1.14 (0.85, 1.52)	0.86 (0.56, 1.32)	1.21 (0.9, 1.62)	0.65 (0.35, 1.21)	2.08 (0.21, 20.22)	**1.3 (1.05, 1.6)**
	**Cis + Doc**	0.79 (0.31, 1.98)	1.03 (0.3, 3.49)	0.6 (0.18, 2)	0.26 (0.05, 1.27)	0.93 (0.39, 2.18)	**Cis + Doc**	1.85 (0.62, 5.52)	1.02 (0.38, 2.71)	0.91 (0.31, 2.69)	0.8 (0.3, 2.15)	0.76 (0.25, 2.36)	1.21 (0.42, 3.51)	1.73 (0.65, 4.6)	0.9 (0.33, 2.48)	0.92 (0.34, 2.52)	1.47 (0.48, 4.45)	1.07 (0.33, 3.4)	1.3 (0.48, 3.5)	0.98 (0.34, 2.78)	1.37 (0.51, 3.71)	0.74 (0.24, 2.31)	2.37 (0.2, 27.89)	1.48 (0.56, 3.92)
	**Cis + Epi**	1.23 (0.67, 2.26)	1.6 (0.6, 4.28)	0.94 (0.37, 2.44)	0.41 (0.1, 1.65)	1.45 (0.84, 2.49)	1.56 (0.56, 4.32)	**Cis + Epi**	**0.55 (0.32, 0.94)**	0.49 (0.24, 1.01)	**0.43 (0.24, 0.78)**	**0.41 (0.18, 0.92)**	0.66 (0.34, 1.26)	0.94 (0.52, 1.68)	**0.49 (0.26, 0.93)**	**0.5 (0.27, 0.94)**	0.79 (0.38, 1.64)	0.58 (0.26, 1.29)	0.7 (0.4, 1.23)	**0.53 (0.28, 0.99)**	0.74 (0.42, 1.32)	**0.4 (0.19, 0.86)**	1.28 (0.12, 13.26)	0.8 (0.49, 1.31)
	**Cis + Flu**	0.92 (0.62, 1.36)	1.2 (0.5, 2.87)	0.71 (0.31, 1.63)	0.31 (0.08, 1.15)	1.08 (0.84, 1.4)	1.17 (0.48, 2.87)	0.75 (0.43, 1.29)	**Cis + Flu**	0.89 (0.53, 1.5)	0.79 (0.56, 1.11)	0.75 (0.39, 1.43)	1.19 (0.74, 1.91)	**1.7 (1.22, 2.35)**	0.88 (0.58, 1.35)	0.9 (0.61, 1.35)	1.44 (0.81, 2.55)	1.04 (0.54, 2.04)	1.27 (0.92, 1.75)	0.96 (0.62, 1.48)	**1.35 (1.01, 1.79)**	0.73 (0.39, 1.36)	2.32 (0.24, 22.86)	**1.45 (1.18, 1.79)**
	**Cis + Flu + Hyd**	0.68 (0.38, 1.24)	0.89 (0.33, 2.38)	0.53 (0.2, 1.36)	**0.23 (0.06, 0.92)**	0.81 (0.48, 1.34)	0.87 (0.32, 2.36)	0.56 (0.27, 1.13)	0.74 (0.45, 1.23)	**Cis + Flu + Hyd**	0.88 (0.49, 1.6)	0.84 (0.37, 1.88)	1.34 (0.68, 2.64)	**1.91 (1.07, 3.41)**	0.99 (0.52, 1.88)	1.02 (0.55, 1.9)	1.62 (0.76, 3.43)	1.18 (0.52, 2.68)	1.43 (0.8, 2.55)	1.08 (0.56, 2.07)	1.52 (0.97, 2.36)	0.82 (0.37, 1.8)	2.61 (0.25, 27.03)	1.63 (0.96, 2.77)
	**Cis + Ifo**	**0.71 (0.54, 0.93)**	0.93 (0.38, 2.28)	0.55 (0.23, 1.29)	**0.24 (0.06, 0.9)**	0.84 (0.65, 1.08)	0.9 (0.37, 2.21)	0.58 (0.32, 1.04)	0.77 (0.55, 1.09)	1.04 (0.59, 1.82)	**Cis + Ifo**	0.95 (0.55, 1.65)	1.51 (0.88, 2.59)	**2.16 (1.52, 3.08)**	1.12 (0.72, 1.75)	1.15 (0.75, 1.76)	1.83 (0.98, 3.42)	1.33 (0.65, 2.71)	**1.62 (1.09, 2.38)**	1.22 (0.74, 2.01)	**1.71 (1.16, 2.53)**	0.92 (0.47, 1.81)	2.95 (0.3, 29.2)	**1.85 (1.33, 2.56)**
	**Cis + Ifo + Pac**	0.57 (0.3, 1.09)	0.74 (0.25, 2.18)	0.44 (0.15, 1.24)	**0.19 (0.04, 0.82)**	0.67 (0.35, 1.28)	0.72 (0.25, 2.12)	0.46 (0.2, 1.07)	0.62 (0.31, 1.23)	0.83 (0.37, 1.88)	0.8 (0.44, 1.45)	**Cis + Ifo + Pac**	1.59 (0.74, 3.44)	**2.27 (1.18, 4.37)**	1.18 (0.58, 2.4)	1.21 (0.61, 2.42)	1.92 (0.84, 4.42)	1.4 (0.57, 3.44)	1.7 (0.87, 3.33)	1.28 (0.61, 2.69)	1.8 (0.92, 3.54)	0.97 (0.41, 2.32)	3.11 (0.29, 32.8)	**1.94 (1.03, 3.68)**
	**Cis + Metho- trexate**	1.06 (0.6, 1.87)	1.38 (0.53, 3.59)	0.81 (0.32, 2.04)	0.35 (0.09, 1.4)	1.25 (0.76, 2.05)	1.35 (0.5, 3.63)	0.86 (0.44, 1.69)	1.15 (0.7, 1.89)	1.55 (0.79, 3.03)	1.49 (0.86, 2.56)	1.86 (0.83, 4.16)	**Cis + Metho- trexate**	1.43 (0.84, 2.42)	0.74 (0.41, 1.34)	0.76 (0.43, 1.35)	1.21 (0.61, 2.39)	0.88 (0.41, 1.89)	1.07 (0.64, 1.77)	0.8 (0.45, 1.43)	1.13 (0.68, 1.89)	0.61 (0.29, 1.26)	1.95 (0.19, 19.94)	1.22 (0.8, 1.87)
	**Cis + Mito- lactol**	0.77 (0.5, 1.19)	1.01 (0.4, 2.52)	0.59 (0.25, 1.43)	**0.26 (0.07, 0.99)**	0.91 (0.69, 1.2)	0.98 (0.4, 2.42)	0.63 (0.34, 1.16)	0.84 (0.58, 1.23)	1.13 (0.63, 2.02)	1.09 (0.75, 1.58)	1.36 (0.67, 2.74)	0.73 (0.41, 1.29)	**Cis + Mito- lactol**	**0.52 (0.34, 0.79)**	**0.53 (0.36, 0.79)**	0.85 (0.46, 1.57)	0.62 (0.3, 1.25)	0.75 (0.52, 1.08)	**0.56 (0.35, 0.92)**	0.79 (0.55, 1.15)	**0.43 (0.22, 0.83)**	1.37 (0.14, 13.46)	0.85 (0.63, 1.16)
	**Cis + Pac**	0.69 (0.44, 1.07)	0.9 (0.36, 2.25)	0.53 (0.22, 1.28)	**0.23 (0.06, 0.89)**	0.81 (0.6, 1.09)	0.87 (0.35, 2.17)	0.56 (0.3, 1.04)	0.75 (0.51, 1.1)	1.01 (0.56, 1.81)	0.97 (0.66, 1.43)	1.21 (0.6, 2.45)	0.65 (0.36, 1.16)	0.89 (0.59, 1.33)	**Cis + Pac**	1.03 (0.63, 1.66)	1.63 (0.83, 3.2)	1.18 (0.56, 2.52)	1.44 (0.91, 2.28)	1.09 (0.62, 1.9)	1.53 (0.96, 2.42)	0.82 (0.4, 1.69)	2.63 (0.28, 24.89)	**1.64 (1.09, 2.48)**
	**Cis + Topo- tecan**	0.84 (0.56, 1.27)	1.1 (0.44, 2.71)	0.65 (0.27, 1.54)	0.28 (0.07, 1.07)	0.99 (0.78, 1.26)	1.07 (0.44, 2.61)	0.68 (0.38, 1.24)	0.91 (0.64, 1.3)	1.23 (0.7, 2.16)	1.18 (0.83, 1.68)	1.48 (0.74, 2.94)	0.79 (0.46, 1.38)	1.09 (0.75, 1.57)	1.22 (0.83, 1.79)	**Cis + Topo- tecan**	1.59 (0.82, 3.07)	1.15 (0.55, 2.43)	1.4 (0.91, 2.17)	1.06 (0.62, 1.82)	1.49 (0.96, 2.31)	0.8 (0.4, 1.62)	2.57 (0.26, 25.55)	**1.6 (1.09, 2.36)**
	**Cis + Vcr**	1.36 (0.73, 2.54)	1.78 (0.66, 4.77)	1.05 (0.4, 2.72)	0.46 (0.11, 1.84)	1.6 (0.92, 2.8)	1.73 (0.62, 4.82)	1.11 (0.54, 2.27)	1.48 (0.85, 2.58)	1.99 (0.97, 4.08)	**1.91 (1.05, 3.48)**	2.39 (1.03, 5.56)	1.29 (0.65, 2.55)	1.76 (0.95, 3.28)	**1.98 (1.05, 3.71)**	1.62 (0.88, 2.97)	**Cis + Vcr**	0.73 (0.32, 1.67)	0.88 (0.49, 1.61)	0.67 (0.35, 1.28)	0.94 (0.51, 1.72)	0.51 (0.23, 1.12)	1.62 (0.15, 16.89)	1.01 (0.59, 1.72)
	**Epi**	1.05 (0.48, 2.26)	1.37 (0.46, 4.05)	0.8 (0.28, 2.32)	0.35 (0.08, 1.52)	1.23 (0.6, 2.53)	1.33 (0.43, 4.08)	0.85 (0.36, 1.99)	1.14 (0.55, 2.33)	1.53 (0.65, 3.58)	1.47 (0.69, 3.12)	1.84 (0.7, 4.79)	0.99 (0.43, 2.25)	1.35 (0.63, 2.93)	1.52 (0.7, 3.31)	1.24 (0.58, 2.66)	0.77 (0.33, 1.81)	**Epi**	1.21 (0.61, 2.42)	0.92 (0.44, 1.92)	1.29 (0.64, 2.58)	0.69 (0.29, 1.65)	2.22 (0.21, 23.8)	1.39 (0.74, 2.62)
	**Flu**	1 (0.63, 1.58)	1.3 (0.52, 3.25)	0.77 (0.32, 1.84)	0.33 (0.09, 1.28)	1.17 (0.83, 1.66)	1.27 (0.5, 3.19)	0.81 (0.44, 1.5)	1.08 (0.73, 1.61)	1.46 (0.8, 2.64)	1.4 (0.92, 2.13)	1.75 (0.85, 3.62)	0.94 (0.53, 1.67)	1.29 (0.83, 2.01)	1.45 (0.92, 2.28)	1.18 (0.77, 1.81)	0.73 (0.39, 1.37)	0.95 (0.44, 2.06)	**Flu**	0.75 (0.47, 1.2)	1.06 (0.73, 1.54)	0.57 (0.3, 1.09)	1.83 (0.18, 18.13)	1.14 (0.87, 1.5)
	**Flu + MitoC**	0.69 (0.37, 1.3)	0.9 (0.34, 2.44)	0.53 (0.2, 1.39)	0.23 (0.06, 0.94)	0.82 (0.47, 1.43)	0.88 (0.32, 2.46)	0.56 (0.27, 1.16)	0.75 (0.43, 1.32)	1.01 (0.49, 2.09)	0.97 (0.53, 1.78)	1.22 (0.52, 2.84)	0.66 (0.33, 1.3)	0.9 (0.48, 1.68)	1.01 (0.53, 1.9)	0.82 (0.45, 1.52)	0.51 (0.25, 1.06)	0.66 (0.28, 1.57)	0.7 (0.37, 1.31)	**Flu + MitoC**	1.41 (0.87, 2.26)	0.76 (0.38, 1.53)	2.42 (0.24, 24.57)	**1.52 (1.04, 2.21)**
	**Hyd**	0.99 (0.66, 1.5)	1.29 (0.53, 3.14)	0.76 (0.33, 1.78)	0.33 (0.09, 1.25)	1.17 (0.88, 1.54)	1.26 (0.51, 3.11)	0.81 (0.46, 1.42)	1.08 (0.81, 1.42)	1.45 (0.95, 2.21)	1.39 (0.97, 2.01)	1.74 (0.87, 3.49)	0.94 (0.56, 1.58)	1.28 (0.86, 1.9)	1.44 (0.96, 2.16)	1.18 (0.81, 1.71)	0.73 (0.41, 1.3)	0.95 (0.45, 1.98)	1 (0.65, 1.52)	1.43 (0.8, 2.56)	**Hyd**	0.54 (0.28, 1.04)	1.72 (0.17, 17.1)	1.08 (0.81, 1.44)
	**MitoC**	0.77 (0.4, 1.49)	1 (0.36, 2.76)	0.59 (0.22, 1.57)	0.26 (0.06, 1.05)	0.9 (0.49, 1.65)	0.97 (0.34, 2.78)	0.62 (0.29, 1.32)	0.83 (0.46, 1.52)	1.12 (0.53, 2.38)	1.08 (0.57, 2.04)	1.35 (0.56, 3.22)	0.72 (0.35, 1.49)	0.99 (0.51, 1.92)	1.11 (0.57, 2.18)	0.91 (0.48, 1.74)	0.56 (0.26, 1.2)	0.73 (0.3, 1.78)	0.77 (0.4, 1.5)	1.11 (0.51, 2.38)	0.77 (0.41, 1.44)	**MitoC**	3.2 (0.3, 33.87)	**2 (1.11, 3.61)**
	**Nedaplatin + Pac**	2.3 (0.16, 33.17)	2.99 (0.18, 48.73)	1.76 (0.11, 28.36)	0.77 (0.04, 14.79)	2.7 (0.19, 38.22)	2.92 (0.18, 47.27)	1.87 (0.12, 27.95)	2.49 (0.17, 35.72)	3.35 (0.23, 49.82)	3.22 (0.23, 46.2)	4.03 (0.26, 61.63)	2.17 (0.15, 32.15)	2.97 (0.21, 42.61)	3.33 (0.24, 46.43)	2.73 (0.19, 39.04)	1.68 (0.11, 25.26)	2.19 (0.14, 34.18)	2.3 (0.16, 33.36)	3.31 (0.22, 49.69)	2.31 (0.16, 33.24)	2.99 (0.2, 45.34)	**Nedaplatin + Pac**	0.62 (0.06, 6.14)
	**Placebo**	1.22 (0.86, 1.73)	1.59 (0.68, 3.69)	0.93 (0.42, 2.09)	0.41 (0.11, 1.49)	**1.43 (1.15, 1.78)**	1.55 (0.64, 3.75)	0.99 (0.6, 1.63)	**1.32 (1.07, 1.64)**	**1.78 (1.08, 2.93)**	**1.71 (1.26, 2.33)**	**2.14 (1.1, 4.17)**	1.15 (0.73, 1.8)	**1.57 (1.11, 2.23)**	**1.77 (1.23, 2.54)**	**1.45 (1.04, 2)**	0.89 (0.53, 1.49)	1.16 (0.58, 2.31)	1.22 (0.86, 1.75)	**1.75 (1.04, 2.95)**	1.23 (0.94, 1.6)	1.59 (0.9, 2.78)	0.53 (0.04, 7.57)	**Placebo**

**Table 4:** Mixed evidence of 5-year overall survival and recurrence-free survival for different treatments

5-year RFS																		
		Ble + Cis + Ifo	Ble + Cis + MitoC + Vbl	Ble + Cis + Vbl	Ble + Cis + Vcr	Cis	Cis + Doc	Cis + Epi	Cis + Flu	Cis + Flu + Hyd	Cis + Metho- trexate	Cis + Vcr	Epi	Flu	Flu + MitoC	Hyd	MitoC	Placebo
**5-year OS**	**Ble + Cis + Ifo**	**Ble + Cis + Ifo**																
	**Ble + Cis + MitoC + Vbl**	0.72 (0.28, 1.85)	**Ble + Cis + MitoC + Vbl**															
	**Ble + Cis + Vbl**	1.3 (0.53, 3.2)	1.79 (0.59, 5.42)	**Ble + Cis + Vbl**	1.05 (0.5, 2.22)	0.92 (0.49, 1.72)	0.59 (0.26, 1.32)	1.48 (0.69, 3.16)	0.81 (0.43, 1.51)	0.66 (0.3, 1.44)	0.95 (0.46, 1.95)	1.14 (0.52, 2.47)	1.02 (0.32, 3.23)	1.04 (0.55, 1.98)	0.82 (0.41, 1.65)	1.04 (0.54, 2.01)	0.47 (0.21, 1.07)	1.18 (0.66, 2.12)
	**Ble + Cis + Vcr**	3.03 (0.99, 9.32)	**4.19 (1.15, 15.24)**	2.34 (0.66, 8.29)	**Ble + Cis + Vcr**	0.87 (0.52, 1.48)	0.56 (0.27, 1.16)	1.41 (0.72, 2.76)	0.77 (0.46, 1.29)	0.62 (0.31, 1.26)	0.9 (0.48, 1.69)	1.08 (0.54, 2.16)	0.97 (0.32, 2.91)	0.99 (0.57, 1.7)	0.78 (0.42, 1.43)	0.99 (0.56, 1.73)	**0.45 (0.21, 0.94)**	1.12 (0.71, 1.79)
	**Cis**	0.8 (0.47, 1.36)	1.1 (0.48, 2.53)	0.62 (0.28, 1.36)	**0.26 (0.09, 0.74)**	**Cis**	0.64 (0.39, 1.06)	1.61 (0.94, 2.76)	0.88 (0.65, 1.19)	0.71 (0.41, 1.24)	1.03 (0.63, 1.68)	1.24 (0.7, 2.18)	1.11 (0.4, 3.09)	1.13 (0.83, 1.53)	0.89 (0.56, 1.4)	1.13 (0.8, 1.6)	**0.51 (0.28, 0.96)**	**1.29 (1.01, 1.63)**
	**Cis + Doc**	0.52 (0.25, 1.09)	0.72 (0.27, 1.9)	0.4 (0.16, 1.03)	**0.17 (0.05, 0.54)**	0.65 (0.39, 1.08)	**Cis + Doc**	2.51 (1.2, 5.24)	1.37 (0.77, 2.46)	1.11 (0.53, 2.35)	1.61 (0.8, 3.23)	1.93 (0.9, 4.1)	1.73 (0.55, 5.41)	1.76 (0.98, 3.16)	1.38 (0.7, 2.73)	1.76 (0.96, 3.24)	0.8 (0.36, 1.78)	**2.01 (1.15, 3.49)**
	**Cis + Epi**	1.18 (0.6, 2.33)	1.63 (0.64, 4.15)	0.91 (0.37, 2.24)	0.39 (0.13, 1.19)	1.48 (0.88, 2.51)	**2.28 (1.1, 4.75)**	**Cis + Epi**	**0.55 (0.32, 0.94)**	**0.44 (0.22, 0.91)**	0.64 (0.33, 1.22)	0.77 (0.38, 1.56)	0.69 (0.23, 2.09)	0.7 (0.4, 1.23)	0.55 (0.3, 1.03)	0.7 (0.39, 1.25)	**0.32 (0.15, 0.68)**	0.8 (0.49, 1.3)
	**Cis + Flu**	0.89 (0.53, 1.5)	1.23 (0.54, 2.81)	0.69 (0.31, 1.51)	**0.29 (0.1, 0.82)**	1.11 (0.85, 1.46)	1.71 (0.96, 3.06)	0.75 (0.45, 1.26)	**Cis + Flu**	0.81 (0.47, 1.39)	1.17 (0.72, 1.89)	1.4 (0.8, 2.46)	1.26 (0.45, 3.5)	1.28 (0.91, 1.81)	1.01 (0.64, 1.58)	1.28 (0.93, 1.77)	0.58 (0.32, 1.08)	**1.46 (1.17, 1.83)**
	**Cis + Flu + Hyd**	0.64 (0.33, 1.23)	0.88 (0.35, 2.21)	0.49 (0.2, 1.19)	**0.21 (0.07, 0.64)**	0.79 (0.5, 1.27)	1.22 (0.61, 2.45)	0.54 (0.28, 1.04)	0.71 (0.45, 1.13)	**Cis + Flu + Hyd**	1.44 (0.73, 2.86)	1.73 (0.83, 3.64)	1.55 (0.5, 4.82)	1.58 (0.88, 2.86)	1.25 (0.64, 2.41)	**1.59 (1.03, 2.45)**	0.72 (0.33, 1.58)	**1.81 (1.06, 3.08)**
	**Cis + Metho- trexate**	0.95 (0.51, 1.78)	1.32 (0.54, 3.22)	0.73 (0.31, 1.73)	**0.31 (0.11, 0.93)**	1.19 (0.76, 1.88)	1.84 (0.93, 3.64)	0.81 (0.43, 1.49)	1.07 (0.69, 1.67)	1.5 (0.82, 2.74)	**Cis + Metho- trexate**	1.2 (0.61, 2.34)	1.07 (0.36, 3.19)	1.1 (0.66, 1.82)	0.86 (0.48, 1.54)	1.1 (0.65, 1.86)	0.5 (0.24, 1.02)	1.25 (0.82, 1.92)
	**Cis + Vcr**	1.26 (0.65, 2.48)	1.75 (0.69, 4.42)	0.97 (0.4, 2.38)	0.42 (0.14, 1.27)	1.58 (0.94, 2.66)	**2.44 (1.18, 5.05)**	1.07 (0.55, 2.08)	1.42 (0.85, 2.37)	**1.99 (1.04, 3.83)**	1.33 (0.72, 2.44)	**Cis + Vcr**	0.9 (0.29, 2.76)	0.91 (0.51, 1.64)	0.72 (0.38, 1.37)	0.92 (0.5, 1.67)	**0.42 (0.19, 0.9)**	1.04 (0.62, 1.74)
	**Epi**	0.92 (0.43, 1.96)	1.27 (0.47, 3.42)	0.71 (0.27, 1.85)	**0.3 (0.09, 0.97)**	1.15 (0.62, 2.14)	1.77 (0.79, 3.97)	0.78 (0.37, 1.65)	1.03 (0.56, 1.91)	1.45 (0.69, 3.03)	0.96 (0.48, 1.95)	0.73 (0.34, 1.53)	**Epi**	1.02 (0.36, 2.88)	0.8 (0.27, 2.35)	1.02 (0.36, 2.91)	0.47 (0.15, 1.47)	1.16 (0.43, 3.16)
	**Flu**	0.95 (0.52, 1.71)	1.31 (0.55, 3.12)	0.73 (0.32, 1.68)	**0.31 (0.11, 0.91)**	1.18 (0.85, 1.64)	1.82 (0.99, 3.35)	0.8 (0.44, 1.44)	1.06 (0.72, 1.56)	1.49 (0.86, 2.58)	0.99 (0.59, 1.67)	0.75 (0.42, 1.34)	1.03 (0.52, 2.02)	**Flu**	0.79 (0.49, 1.27)	1 (0.67, 1.5)	**0.46 (0.24, 0.86)**	1.14 (0.86, 1.5)
	**Flu + MitoC**	0.69 (0.35, 1.37)	0.95 (0.37, 2.43)	0.53 (0.22, 1.31)	**0.23 (0.07, 0.7)**	0.86 (0.51, 1.47)	1.33 (0.63, 2.79)	0.58 (0.29, 1.15)	0.78 (0.46, 1.31)	1.09 (0.56, 2.12)	0.72 (0.39, 1.35)	0.55 (0.28, 1.07)	0.75 (0.35, 1.6)	0.73 (0.4, 1.32)	**Flu + MitoC**	1.27 (0.77, 2.1)	0.58 (0.29, 1.16)	1.45 (0.98, 2.14)
	**Hyd**	0.98 (0.57, 1.69)	1.35 (0.58, 3.13)	0.75 (0.34, 1.68)	**0.32 (0.11, 0.91)**	1.22 (0.93, 1.6)	**1.88 (1.06, 3.36)**	0.83 (0.48, 1.42)	1.1 (0.84, 1.43)	**1.54 (1.05, 2.25)**	1.02 (0.64, 1.64)	0.77 (0.45, 1.32)	1.06 (0.56, 2)	1.03 (0.69, 1.53)	1.42 (0.82, 2.45)	**Hyd**	**0.46 (0.24, 0.87)**	1.14 (0.83, 1.55)
	**MitoC**	0.74 (0.34, 1.59)	1.02 (0.37, 2.77)	0.57 (0.21, 1.5)	**0.24 (0.07, 0.79)**	0.92 (0.48, 1.75)	1.42 (0.62, 3.22)	0.62 (0.29, 1.34)	0.83 (0.44, 1.56)	1.16 (0.54, 2.47)	0.77 (0.38, 1.58)	0.58 (0.27, 1.25)	0.8 (0.35, 1.85)	0.78 (0.39, 1.55)	1.07 (0.49, 2.32)	0.75 (0.39, 1.45)	**MitoC**	**2.5 (1.41, 4.43)**
	**Placebo**	1.15 (0.71, 1.86)	1.59 (0.71, 3.54)	0.88 (0.41, 1.89)	0.38 (0.14, 1.04)	**1.44 (1.15, 1.8)**	**2.22 (1.27, 3.87)**	0.97 (0.6, 1.56)	**1.29 (1.05, 1.59)**	**1.81 (1.15, 2.86)**	1.2 (0.81, 1.79)	0.91 (0.57, 1.45)	1.25 (0.7, 2.24)	1.21 (0.86, 1.71)	**1.67 (1.02, 2.71)**	1.18 (0.91, 1.52)	1.56 (0.85, 2.86)	**Placebo**

**Table 5:** *P*-score for each treatment under the outcomes of overall survival and recurrence-free survival

Regimen	Overall survival			Recurrence-free survival		
	1y	3y	5y	1y	3y	5y
**Ble + Cis + Ifo**	0.438	0.405	0.429	0.648	**0.832**	-
**Ble + Cis + MitoC + Vbl**	**0.692**	0.638	0.686	-	-	-
**Ble + Cis + Vbl**	0.529	0.274	0.259	0.556	0.428	0.429
**Ble + Cis + Vcr**	0.348	0.070	0.024	0.307	0.374	0.373
**Cis**	0.559	0.584	0.680	0.594	0.471	0.524
**Cis + Doc**	0.453	0.618	**0.918**	0.359	0.576	**0.857**
**Cis + Epi**	0.573	0.261	0.277	0.200	0.119	0.101
**Cis + Flu**	0.493	0.494	0.555	**0.716**	0.598	0.674
**Cis + Flu + Hyd**	**0.670**	0.768	**0.834**	0.623	0.688	**0.801**
**Cis + Ifo**	0.619	**0.771**	-	**0.690**	**0.810**	-
**Cis + Ifo + Pac**	0.582	**0.856**	-	0.612	0.799	-
**Cis + Methotrexate**	0.442	0.373	0.474	0.417	0.422	0.483
**Cis + Mitolactol**	0.438	0.685	-	0.466	0.118	-
**Cis + Pac**	0.389	**0.791**	-	0.550	0.706	-
**Cis + Topotecan**	0.423	0.593	-	0.483	0.686	-
**Cis + Vcr**	0.484	0.198	0.225	0.304	0.270	0.303
**Epi**	0.531	0.406	0.511	0.577	0.541	0.430
**Flu**	0.305	0.414	0.477	0.369	0.348	0.373
**Flu + MitoC**	0.463	0.750	**0.767**	0.495	0.632	0.641
**Hyd**	**0.668**	0.409	0.436	0.583	0.288	0.367
**MitoC**	0.563	0.664	0.700	**0.653**	**0.820**	**0.940**
**Nedaplatin + Pac**	-	0.264	-	-	0.264	-
**Placebo**	0.338	0.214	0.248	0.296	0.212	0.203

### Recurrence-free survival (RFS)

As to 3-year and 5-year RFS, another primary outcomes, were evaluated in 21 and 14 trials respectively. According to Tables 2–4, nine of them were noticeably better than treatments without chemotherapy for 3-year RFS, as mitomycin C (HR = 2, 95% CI: 1.11-3.61), cisplatin+ifosfamide+paclitaxel (HR = 1.94, 95% CrI: 1.03-3.68), bleomycin+cisplatin+ifosfamide (HR = 1.91, 95% CrI: 1.31-2.8), cisplatin+ifosfamide (HR = 1.85, 95% CrI: 1.33-2.56), cisplatin+paclitaxel (HR = 1.64, 95% CrI: 1.03-3.68), cisplatin+topotecan (HR = 1.6, 95% CrI: 1.09-2.36), fluorouracil+mitomycin C (HR = 1.52, 95% CrI: 1.04-2.21), cisplatin+fluorouracil (HR = 1.45, 95% CrI: 1.18-1.79), cisplatin (HR = 1.3, 95% CrI: 1.05-1.6). Besides, mitomycin C (HR = 2.5, 95% CI: 1.41-4.43), cisplatin+docetaxel (HR = -2.01, 95% CrI: 1.15-3.49), cisplatin+fluorouracil+hydroxyurea (HR = 1.81, 95% CrI: 1.06-3.08), cisplatin+fluorouracil (HR = 1.46, 95% CrI: 1.17-1.83), cisplatin (HR = 1.29, 95% CrI: 1.01-1.63) possessed obvious strength in 5 year RFS. In Table 5, ranking analysis showed that mitomycin C had excellent performance in both short term and long term RFS. Cisplatin+ifosfamide was also efficacious in 1-year and 3-year RFS and since the lack of 5-year RFS, we could not estimate its long term efficacy. Cisplatin+docetaxel and cisplatin+ fluorouracil+hydroxyurea had advantage in improving 5-year RFS, which verified their outstanding efficacy in long-term RFS.

### Recurrence

**Figure 2:**
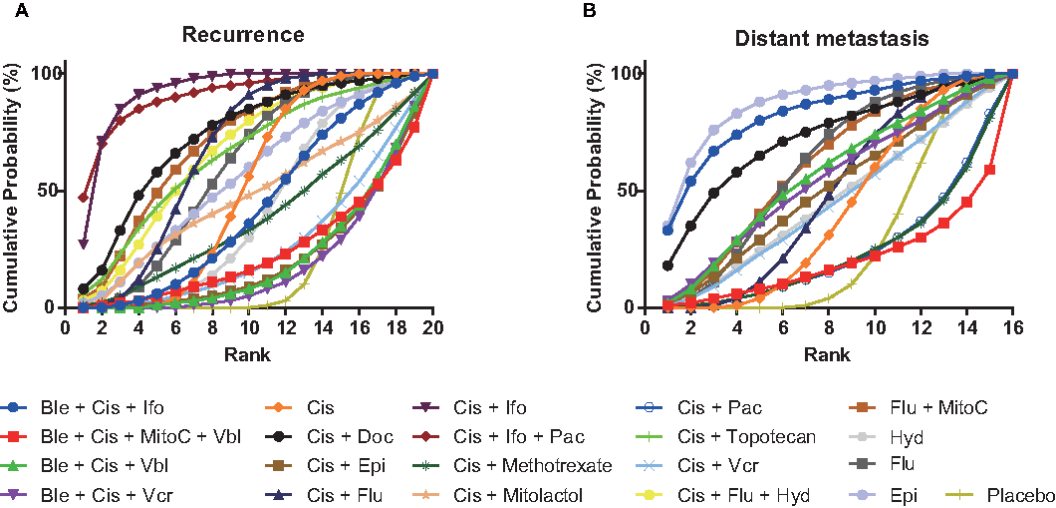
Cumulative ranking probability curves for recurrence and distant metastasis **(A)** recurrence; **(B)** distant metastasis.

In terms of the secondary outcome of recurrence, 19 chemotherapy strategies were compared with placebo in Table 6. Cisplatin+ifosfamide+paclitaxel (OR = 0.15, 95% CrI: 0.03-0.77), cisplatin+ ifosfamide (OR = 0.17, 95% CrI: 0.05-0.53), cisplatin+fluorouracil (OR = 0.45, 95% CrI: 0.29-0.71), fluorouracil (OR = 0.51, 95% CrI: 0.26-0.96), and cisplatin (OR = 0.59, 95% CrI: 0.37-0.93) were outstanding among them. Based on their SUCRA illustrated in Figure 2 and Table 8, cisplatin+ifosfamide (0.93) was the optimal combination, cisplatin+ifosfamide+paclitaxel (0.91) was the second, and the third one was cisplatin+docetaxel (0.74).

**Table 6:** Mixed evidence of recurrence for different treatments

	Ble+Cis+Ifo	Ble+Cis+MitoC+Vbl	Ble+Cis+Vbl	Ble+Cis+Vcr	Cis	Cis+Doc	Cis+Epi	Cis+Flu	Cis+Ifo	Cis+Ifo+Pac	Cis+Methotrexate	Cis+Mitolactol	Cis+Topotecan	Cis+Vcr	Epi	Flu	Cis+Flu+Hyd	Flu+MitoC	Hyd	Placebo
**Ble+Cis+Ifo**	**Ble+Cis+Ifo**	1.73 (0.33, 8.40)	1.68 (0.48, 5.90)	1.77 (0.53, 5.58)	0.85 (0.33, 2.06)	0.51 (0.11, 2.12)	1.72 (0.44, 6.58)	0.65 (0.25, 1.59)	0.24 (0.07, 0.74)	0.22 (0.04, 1.09)	1.18 (0.23, 5.85)	0.91 (0.17, 5.27)	0.61 (0.13, 2.89)	1.50 (0.37, 5.48)	0.77 (0.18, 2.93)	0.73 (0.25, 2.00)	0.62 (0.16, 2.13)	0.56 (0.15, 1.92)	1.02 (0.33, 2.68)	1.43 (0.61, 3.20)
**Ble+Cis+MitoC+Vbl**	0.58 (0.12, 2.99)	**Ble+Cis+MitoC+Vbl**	0.98 (0.19, 5.19)	1.04 (0.21, 5.24)	0.49 (0.11, 2.13)	0.30 (0.05, 1.81)	1.01 (0.17, 5.95)	0.38 (0.09, 1.61)	0.14 (0.02, 0.86)	0.13 (0.02, 1.08)	0.69 (0.10, 4.76)	0.53 (0.07, 5.08)	0.36 (0.05, 2.47)	0.87 (0.16, 4.95)	0.44 (0.08, 2.62)	0.42 (0.09, 1.93)	0.36 (0.07, 1.90)	0.33 (0.06, 1.74)	0.59 (0.13, 2.53)	0.84 (0.21, 3.32)
**Ble+Cis+Vbl**	0.60 (0.17, 2.10)	1.02 (0.19, 5.34)	**Ble+Cis+Vbl**	1.06 (0.29, 3.76)	0.50 (0.17, 1.46)	0.30 (0.06, 1.42)	1.02 (0.25, 4.33)	0.38 (0.13, 1.10)	0.14 (0.03, 0.65)	0.13 (0.02, 0.87)	0.70 (0.13, 3.75)	0.54 (0.09, 3.82)	0.37 (0.07, 1.85)	0.89 (0.20, 3.71)	0.46 (0.10, 1.99)	0.43 (0.13, 1.36)	0.37 (0.09, 1.44)	0.33 (0.08, 1.28)	0.60 (0.19, 1.78)	0.85 (0.33, 2.19)
**Ble+Cis+Vcr**	0.56 (0.18, 1.88)	0.97 (0.19, 4.83)	0.95 (0.27, 3.39)	**Ble+Cis+Vcr**	0.48 (0.18, 1.25)	0.29 (0.06, 1.27)	0.97 (0.25, 3.76)	0.37 (0.14, 0.94)	0.14 (0.03, 0.57)	0.12 (0.02, 0.78)	0.67 (0.13, 3.31)	0.51 (0.09, 3.56)	0.34 (0.07, 1.69)	0.84 (0.21, 3.27)	0.43 (0.10, 1.70)	0.41 (0.14, 1.17)	0.35 (0.09, 1.27)	0.31 (0.09, 1.12)	0.57 (0.19, 1.53)	0.81 (0.35, 1.87)
**Cis**	1.18 (0.48, 3.05)	2.03 (0.47, 8.74)	1.99 (0.68, 5.73)	2.10 (0.80, 5.45)	**Cis**	0.60 (0.19, 1.86)	2.05 (0.64, 6.67)	0.76 (0.44, 1.31)	0.29 (0.09, 0.89)	0.26 (0.05, 1.31)	1.40 (0.32, 6.03)	1.07 (0.23, 6.03)	0.73 (0.21, 2.59)	1.77 (0.55, 5.67)	0.91 (0.27, 3.02)	0.85 (0.42, 1.71)	0.74 (0.27, 1.84)	0.66 (0.23, 1.89)	1.21 (0.59, 2.18)	1.69 (1.06, 2.68)
**Cis+Doc**	1.97 (0.47, 8.71)	3.38 (0.55, 21.43)	3.35 (0.71, 16.05)	3.51 (0.79, 15.93)	1.68 (0.54, 5.27)	**Cis+Doc**	3.39 (0.66, 18.11)	1.28 (0.36, 4.55)	0.48 (0.10, 2.47)	0.44 (0.06, 3.23)	2.34 (0.37, 14.68)	1.81 (0.26, 14.25)	1.22 (0.22, 6.64)	2.97 (0.57, 15.12)	1.52 (0.29, 8.02)	1.44 (0.37, 5.51)	1.23 (0.26, 5.29)	1.12 (0.23, 5.28)	2.01 (0.50, 7.15)	2.84 (0.82, 9.70)
**Cis+Epi**	0.58 (0.15, 2.27)	0.99 (0.17, 5.72)	0.98 (0.23, 4.08)	1.03 (0.27, 4.03)	0.49 (0.15, 1.55)	0.29 (0.06, 1.51)	**Cis+Epi**	0.38 (0.11, 1.19)	0.14 (0.03, 0.68)	0.13 (0.02, 0.91)	0.68 (0.12, 3.88)	0.53 (0.08, 3.96)	0.36 (0.06, 1.93)	0.87 (0.19, 3.85)	0.45 (0.09, 2.03)	0.42 (0.11, 1.45)	0.36 (0.08, 1.52)	0.33 (0.07, 1.36)	0.59 (0.16, 1.89)	0.83 (0.28, 2.38)
**Cis+Flu**	1.54 (0.63, 4.03)	2.65 (0.62, 11.16)	2.61 (0.91, 7.46)	**2.73 (1.07, 7.22)**	1.31 (0.76, 2.26)	0.78 (0.22, 2.77)	2.66 (0.84, 8.77)	**Cis+Flu**	0.38 (0.11, 1.24)	0.34 (0.07, 1.83)	1.83 (0.42, 7.83)	1.41 (0.28, 8.31)	0.95 (0.24, 3.65)	2.30 (0.72, 7.41)	1.18 (0.36, 3.93)	1.11 (0.51, 2.43)	0.96 (0.33, 2.58)	0.86 (0.29, 2.49)	1.57 (0.77, 2.83)	2.21 (1.40, 3.53)
**Cis+Ifo**	**4.08 (1.35, 13.49)**	**7.12 (1.17, 42.43)**	**6.91 (1.55, 31.52)**	**7.30 (1.74, 30.66)**	**3.47 (1.13, 11.09)**	2.06 (0.41, 10.46)	**7.10 (1.48, 35.27)**	2.65 (0.81, 9.06)	**Cis+Ifo**	0.90 (0.30, 2.89)	4.87 (0.78, 30.22)	3.71 (0.92, 18.13)	2.51 (0.46, 13.93)	6.09 (1.28, 29.86)	3.15 (0.62, 15.87)	2.96 (0.81, 11.07)	2.53 (0.57, 10.86)	2.29 (0.51, 10.21)	4.14 (1.12, 14.63)	5.86 (1.90, 19.01)
**Cis+Ifo+Pac**	4.53 (0.92, 22.95)	7.72 (0.93, 64.19)	**7.58 (1.15, 48.69)**	**8.00 (1.27, 47.80)**	3.81 (0.76, 18.89)	2.28 (0.31, 16.11)	**7.78 (1.09, 54.27)**	2.92 (0.55, 14.99)	1.11 (0.35, 3.31)	**Cis+Ifo+Pac**	5.36 (0.61, 44.30)	4.10 (0.69, 27.85)	2.75 (0.35, 20.72)	6.67 (0.94, 47.95)	3.43 (0.48, 25.04)	3.25 (0.58, 18.35)	2.79 (0.43, 16.82)	2.52 (0.39, 16.28)	4.61 (0.79, 23.56)	6.47 (1.28, 31.85)
**Cis+Methotrexate**	0.85 (0.17, 4.28)	1.45 (0.21, 10.09)	1.42 (0.27, 7.68)	1.49 (0.30, 7.58)	0.72 (0.17, 3.11)	0.43 (0.07, 2.67)	1.46 (0.26, 8.52)	0.55 (0.13, 2.36)	0.21 (0.03, 1.28)	0.19 (0.02, 1.63)	**Cis+Methotrexate**	0.78 (0.10, 6.92)	0.51 (0.08, 3.57)	1.26 (0.22, 7.11)	0.65 (0.11, 3.91)	0.61 (0.13, 2.83)	0.52 (0.09, 2.79)	0.47 (0.09, 2.50)	0.86 (0.19, 3.69)	1.21 (0.31, 4.82)
**Cis+Mitolactol**	1.09 (0.19, 5.90)	1.90 (0.20, 15.11)	1.85 (0.26, 11.69)	1.97 (0.28, 11.13)	0.93 (0.17, 4.32)	0.55 (0.07, 3.78)	1.89 (0.25, 12.86)	0.71 (0.12, 3.55)	0.27 (0.06, 1.09)	0.24 (0.04, 1.46)	1.29 (0.14, 10.22)	**Cis+Mitolactol**	0.67 (0.08, 4.90)	1.64 (0.21, 10.64)	0.84 (0.10, 5.77)	0.79 (0.12, 4.18)	0.68 (0.09, 4.06)	0.62 (0.08, 3.85)	1.11 (0.17, 5.60)	1.58 (0.27, 7.52)
**Cis+Topotecan**	1.63 (0.35, 7.93)	2.79 (0.41, 19.03)	2.74 (0.54, 14.35)	2.90 (0.59, 14.27)	1.37 (0.39, 4.81)	0.82 (0.15, 4.47)	2.80 (0.52, 15.94)	1.05 (0.27, 4.13)	0.40 (0.07, 2.18)	0.36 (0.05, 2.88)	1.94 (0.28, 12.66)	1.50 (0.20, 12.39)	**Cis+Topotecan**	2.41 (0.43, 13.44)	1.25 (0.22, 6.98)	1.18 (0.28, 4.94)	1.01 (0.20, 4.66)	0.90 (0.18, 4.57)	1.65 (0.39, 6.37)	2.33 (0.61, 8.94)
**Cis+Vcr**	0.67 (0.18, 2.70)	1.16 (0.20, 6.39)	1.12 (0.27, 4.91)	1.19 (0.31, 4.66)	0.57 (0.18, 1.82)	0.34 (0.07, 1.75)	1.15 (0.26, 5.20)	0.43 (0.13, 1.39)	0.16 (0.03, 0.78)	0.15 (0.02, 1.06)	0.79 (0.14, 4.46)	0.61 (0.09, 4.77)	0.41 (0.07, 2.32)	**Cis+Vcr**	0.52 (0.11, 2.40)	0.49 (0.14, 1.68)	0.42 (0.09, 1.74)	0.38 (0.09, 1.62)	0.68 (0.19, 2.18)	0.96 (0.33, 2.83)
**Epi**	1.30 (0.34, 5.48)	2.25 (0.38, 13.27)	2.19 (0.50, 9.60)	2.30 (0.59, 9.59)	1.10 (0.33, 3.66)	0.66 (0.12, 3.45)	2.24 (0.49, 10.59)	0.85 (0.25, 2.78)	0.32 (0.06, 1.60)	0.29 (0.04, 2.07)	1.54 (0.26, 9.12)	1.19 (0.17, 9.58)	0.80 (0.14, 4.63)	1.93 (0.42, 9.12)	**Epi**	0.94 (0.26, 3.39)	0.81 (0.18, 3.45)	0.72 (0.16, 3.21)	1.32 (0.36, 4.47)	1.87 (0.62, 5.71)
**Flu**	1.37 (0.50, 4.04)	2.38 (0.52, 11.00)	2.34 (0.73, 7.50)	2.46 (0.85, 7.23)	1.17 (0.59, 2.38)	0.69 (0.18, 2.71)	2.38 (0.69, 8.71)	0.90 (0.41, 1.96)	0.34 (0.09, 1.24)	0.31 (0.05, 1.73)	1.64 (0.35, 7.77)	1.26 (0.24, 8.06)	0.85 (0.20, 3.58)	2.06 (0.59, 7.20)	1.06 (0.30, 3.92)	**Flu**	0.86 (0.26, 2.69)	0.77 (0.29, 2.11)	1.41 (0.56, 3.16)	1.98 (1.03, 3.91)
**Cis+Flu+Hyd**	1.61 (0.47, 6.28)	2.79 (0.53, 15.34)	2.72 (0.69, 11.11)	2.85 (0.79, 11.08)	1.36 (0.54, 3.74)	0.81 (0.19, 3.82)	2.79 (0.66, 12.37)	1.04 (0.39, 3.05)	0.39 (0.09, 1.76)	0.36 (0.06, 2.34)	1.91 (0.36, 10.73)	1.47 (0.25, 10.55)	0.99 (0.21, 5.00)	2.39 (0.57, 10.76)	1.24 (0.29, 5.68)	1.16 (0.37, 3.81)	**Cis+Flu+Hyd**	0.90 (0.24, 3.75)	1.64 (0.61, 4.13)	2.30 (0.88, 6.50)
**Flu+MitoC**	1.78 (0.52, 6.68)	3.07 (0.57, 16.69)	3.02 (0.78, 11.79)	3.18 (0.90, 11.66)	1.51 (0.53, 4.27)	0.90 (0.19, 4.32)	3.06 (0.74, 13.35)	1.16 (0.40, 3.43)	0.44 (0.10, 1.95)	0.40 (0.06, 2.58)	2.11 (0.40, 11.57)	1.62 (0.26, 11.85)	1.11 (0.22, 5.51)	2.66 (0.62, 11.38)	1.38 (0.31, 6.10)	1.29 (0.47, 3.44)	1.11 (0.27, 4.24)	**Flu+MitoC**	1.82 (0.56, 5.36)	2.56 (0.95, 6.80)
**Hyd**	0.98 (0.37, 3.02)	1.69 (0.40, 7.94)	1.67 (0.56, 5.31)	1.75 (0.66, 5.30)	0.83 (0.46, 1.70)	0.50 (0.14, 2.00)	1.69 (0.53, 6.32)	0.64 (0.35, 1.29)	**0.24 (0.07, 0.89)**	0.22 (0.04, 1.27)	1.16 (0.27, 5.40)	0.90 (0.18, 5.81)	0.61 (0.16, 2.58)	1.46 (0.46, 5.29)	0.76 (0.22, 2.80)	0.71 (0.32, 1.79)	0.61 (0.24, 1.63)	0.55 (0.19, 1.79)	**Hyd**	1.41 (0.81, 2.73)
**Placebo**	0.70 (0.31, 1.64)	1.19 (0.30, 4.73)	1.18 (0.46, 3.05)	1.24 (0.54, 2.85)	**0.59 (0.37, 0.94)**	0.35 (0.10, 1.23)	1.20 (0.42, 3.60)	**0.45 (0.28, 0.71)**	**0.17 (0.05, 0.53)**	**0.15 (0.03, 0.78)**	0.83 (0.21, 3.27)	0.63 (0.13, 3.66)	0.43 (0.11, 1.64)	1.04 (0.35, 3.03)	0.54 (0.18, 1.61)	**0.51 (0.26, 0.97)**	0.43 (0.15, 1.13)	0.39 (0.15, 1.05)	0.71 (0.37, 1.24)	**Placebo**

**Table 7:** Mixed evidence of distant metastasis for different treatments

	Ble+Cis+Ifo	Ble+Cis+MitoC+Vbl	Ble+Cis+Vbl	Ble+Cis+Vcr	Cis	Cis+Doc	Cis+Epi	Cis+Flu	Cis+Metho-trexate	Cis+Pac	Cis+Vcr	Epi	Flu	Flu+MitoC	Hyd	Placebo
**Ble+Cis+Ifo**	**Ble+Cis+Ifo**	5.29 (0.65, 51.92)	2.19 (0.38, 14.21)	2.22 (0.34, 16.32)	2.75 (0.59, 15.69)	1.42 (0.15, 14.00)	2.43 (0.40, 17.76)	2.50 (0.55, 14.39)	4.02 (0.66, 28.87)	4.04 (0.65, 29.51)	2.68 (0.45, 18.96)	0.92 (0.14, 6.74)	2.03 (0.40, 12.01)	2.02 (0.36, 12.74)	2.69 (0.43, 22.55)	3.29 (0.79, 16.69)
**Ble+Cis +MitoC+Vbl**	0.19 (0.02, 1.53)	**Ble+Cis +MitoC+Vbl**	0.41 (0.06, 2.64)	0.42 (0.05, 2.88)	0.52 (0.09, 2.70)	0.26 (0.02, 2.58)	0.46 (0.06, 3.16)	0.47 (0.09, 2.57)	0.77 (0.10, 5.07)	0.76 (0.10, 5.47)	0.50 (0.07, 3.30)	0.18 (0.02, 1.28)	0.38 (0.06, 2.14)	0.38 (0.06, 2.39)	0.50 (0.07, 3.95)	0.63 (0.12, 3.03)
**Ble+Cis+Vbl**	0.46 (0.07, 2.65)	2.46 (0.38, 17.09)	**Ble+Cis+Vbl**	1.02 (0.21, 5.11)	1.29 (0.37, 4.25)	0.67 (0.09, 4.65)	1.15 (0.25, 5.13)	1.17 (0.36, 4.06)	1.92 (0.40, 8.31)	1.90 (0.38, 8.67)	1.25 (0.27, 5.50)	0.44 (0.08, 2.07)	0.95 (0.25, 3.35)	0.94 (0.22, 3.78)	1.24 (0.25, 7.02)	1.54 (0.53, 4.32)
**Ble+Cis+Vcr**	0.45 (0.06, 2.97)	2.38 (0.35, 18.82)	0.98 (0.20, 4.79)	**Ble+Cis+Vcr**	1.24 (0.32, 4.73)	0.65 (0.08, 4.98)	1.10 (0.21, 5.91)	1.12 (0.31, 4.80)	1.87 (0.36, 9.48)	1.81 (0.33, 9.93)	1.21 (0.23, 6.07)	0.42 (0.08, 2.27)	0.92 (0.21, 3.68)	0.92 (0.19, 4.26)	1.21 (0.23, 7.69)	1.50 (0.45, 4.99)
**Cis**	0.36 (0.06, 1.68)	1.92 (0.37, 11.15)	0.77 (0.24, 2.70)	0.80 (0.21, 3.10)	**Cis**	0.51 (0.10, 2.46)	0.89 (0.25, 3.24)	0.90 (0.48, 1.92)	1.48 (0.42, 5.18)	1.48 (0.52, 4.15)	0.98 (0.27, 3.29)	0.34 (0.09, 1.27)	0.74 (0.33, 1.57)	0.74 (0.25, 2.11)	0.96 (0.28, 3.88)	1.20 (0.65, 2.16)
**Cis+Doc**	0.70 (0.07, 6.59)	3.81 (0.39, 40.21)	1.48 (0.22, 11.65)	1.53 (0.20, 12.84)	1.95 (0.41, 10.07)	**Cis+Doc**	1.72 (0.23, 13.70)	1.75 (0.34, 11.17)	2.84 (0.38, 23.43)	2.82 (0.44, 19.85)	1.90 (0.25, 14.22)	0.66 (0.08, 5.24)	1.43 (0.24, 8.61)	1.42 (0.21, 9.99)	1.88 (0.27, 16.27)	2.33 (0.43, 13.23)
**Cis+Epi**	0.41 (0.06, 2.53)	2.19 (0.32, 16.23)	0.87 (0.20, 4.08)	0.91 (0.17, 4.67)	1.13 (0.31, 4.02)	0.58 (0.07, 4.32)	**Cis+Epi**	1.02 (0.30, 3.89)	1.66 (0.33, 8.04)	1.66 (0.31, 8.47)	1.09 (0.22, 5.01)	0.39 (0.07, 1.95)	0.83 (0.20, 3.24)	0.83 (0.18, 3.53)	1.09 (0.22, 6.59)	1.35 (0.43, 4.14)
**Cis+Flu**	0.40 (0.07, 1.80)	2.13 (0.39, 11.76)	0.86 (0.25, 2.75)	0.89 (0.21, 3.21)	1.11 (0.52, 2.08)	0.57 (0.09, 2.97)	0.98 (0.26, 3.35)	**Cis+Flu**	1.65 (0.43, 5.40)	1.64 (0.43, 5.31)	1.09 (0.28, 3.38)	0.37 (0.09, 1.36)	0.82 (0.29, 1.84)	0.83 (0.24, 2.29)	1.07 (0.35, 3.31)	1.33 (0.68, 2.23)
**Cis+Metho- trexate**	0.25 (0.03, 1.52)	1.30 (0.20, 9.64)	0.52 (0.12, 2.50)	0.54 (0.11, 2.80)	0.67 (0.19, 2.37)	0.35 (0.04, 2.63)	0.60 (0.12, 3.05)	0.61 (0.19, 2.33)	**Cis+Metho- trexate**	1.00 (0.20, 5.27)	0.66 (0.13, 3.12)	0.23 (0.04, 1.18)	0.50 (0.13, 1.93)	0.50 (0.11, 2.14)	0.65 (0.13, 3.88)	0.82 (0.27, 2.47)
**Cis+Pac**	0.25 (0.03, 1.54)	1.32 (0.18, 10.06)	0.53 (0.12, 2.62)	0.55 (0.10, 3.00)	0.68 (0.24, 1.92)	0.35 (0.05, 2.28)	0.60 (0.12, 3.19)	0.61 (0.19, 2.31)	1.00 (0.19, 5.12)	**Cis+Pac**	0.66 (0.13, 3.25)	0.23 (0.04, 1.24)	0.50 (0.13, 1.83)	0.50 (0.11, 2.16)	0.65 (0.14, 3.76)	0.82 (0.24, 2.66)
**Cis+Vcr**	0.37 (0.05, 2.23)	1.98 (0.30, 14.48)	0.80 (0.18, 3.75)	0.82 (0.16, 4.30)	1.02 (0.30, 3.72)	0.53 (0.07, 4.00)	0.92 (0.20, 4.59)	0.92 (0.30, 3.52)	1.51 (0.32, 7.42)	1.52 (0.31, 7.63)	**Cis+Vcr**	0.35 (0.07, 1.77)	0.76 (0.20, 3.01)	0.76 (0.18, 3.36)	1.00 (0.21, 6.02)	1.24 (0.42, 3.75)
**Epi**	1.08 (0.15, 7.01)	5.65 (0.78, 44.07)	2.29 (0.48, 11.93)	2.36 (0.44, 13.23)	2.93 (0.79, 11.66)	1.51 (0.19, 12.41)	2.59 (0.51, 14.69)	2.67 (0.73, 11.15)	4.36 (0.85, 22.93)	4.29 (0.81, 24.10)	2.89 (0.56, 14.54)	**Epi**	2.16 (0.52, 9.33)	2.15 (0.47, 10.39)	2.83 (0.57, 18.09)	3.55 (1.09, 12.39)
**Flu**	0.49 (0.08, 2.51)	2.61 (0.47, 16.14)	1.06 (0.30, 4.07)	1.09 (0.27, 4.68)	1.36 (0.64, 3.06)	0.70 (0.12, 4.12)	1.21 (0.31, 5.04)	1.22 (0.54, 3.46)	2.01 (0.52, 7.82)	2.00 (0.55, 7.60)	1.32 (0.33, 5.09)	0.46 (0.11, 1.94)	**Flu**	1.00 (0.37, 2.70)	1.29 (0.35, 6.33)	1.63 (0.76, 3.66)
**Flu+MitoC**	0.49 (0.08, 2.81)	2.62 (0.42, 17.61)	1.06 (0.26, 4.63)	1.09 (0.23, 5.20)	1.35 (0.47, 3.99)	0.70 (0.10, 4.70)	1.21 (0.28, 5.56)	1.20 (0.44, 4.12)	1.99 (0.47, 8.80)	2.00 (0.46, 9.11)	1.32 (0.30, 5.52)	0.46 (0.10, 2.15)	1.00 (0.37, 2.70)	**Flu+MitoC**	1.31 (0.30, 7.13)	1.62 (0.63, 4.33)
**Hyd**	0.37 (0.04, 2.33)	2.02 (0.25, 14.72)	0.81 (0.14, 3.99)	0.83 (0.13, 4.42)	1.04 (0.26, 3.52)	0.53 (0.06, 3.70)	0.91 (0.15, 4.54)	0.93 (0.30, 2.83)	1.54 (0.26, 7.51)	1.53 (0.27, 7.27)	1.00 (0.17, 4.77)	0.35 (0.06, 1.77)	0.77 (0.16, 2.87)	0.76 (0.14, 3.30)	**Hyd**	1.24 (0.32, 3.95)
**Placebo**	0.30 (0.06, 1.26)	1.59 (0.33, 8.26)	0.65 (0.23, 1.89)	0.67 (0.20, 2.24)	0.83 (0.46, 1.53)	0.43 (0.08, 2.30)	0.74 (0.24, 2.30)	0.75 (0.45, 1.48)	1.23 (0.40, 3.75)	1.22 (0.38, 4.11)	0.81 (0.27, 2.40)	**0.28 (0.08, 0.91)**	0.61 (0.27, 1.32)	0.62 (0.23, 1.58)	0.81 (0.25, 3.17)	**Placebo**

**Table 8:** SUCRA value for each treatment under the outcomes of recurrence and distant metastasis

	Outcome	
	Recurrence	Distant metastasis
**Ble + Cis + Ifo**	0.43	**0.83**
**Ble + Cis + MitoC + Vbl**	0.24	0.20
**Ble + Cis + Vbl**	0.21	0.56
**Ble + Cis + Vcr**	0.18	0.54
**Cis**	0.52	0.41
**Cis + Doc**	**0.74**	**0.72**
**Cis + Epi**	0.20	0.50
**Cis + Flu**	0.68	0.48
**Cis + Flu + Hyd**	0.67	-
**Cis + Ifo**	**0.93**	-
**Cis + Ifo + Pac**	**0.91**	-
**Cis + Methotrexate**	0.38	0.23
**Cis + Mitolactol**	0.47	-
**Cis + Pac**	-	0.23
**Cis + Topotecan**	0.66	-
**Cis + Vcr**	0.26	0.45
**Epi**	0.56	**0.88**
**Flu**	0.60	0.61
**Flu + MitoC**	0.71	0.61
**Hyd**	0.42	0.45
**Placebo**	0.24	0.28

### Distant metastasis

The potencies of reducing the occurrence of distant metastasis were also estimated, and 15 interventions had data in contrast with placebo, shown in Table 7. Only epirubicin (OR = 0.28, 95% CrI: 0.08-0.91) was significantly excellent in reducing the occurrence of distant metastasis. This was also affirmed in our SUCRA rank probability diagram, seen in Figure 2 and Table 8, since the SUCRA of epirubicin was 0.88, and the following two were bleomycin+cisplatin+ifosfamide (0.83), and cisplatin+docetaxel (0.73).

### Inconsistency test

**Figure 3:**
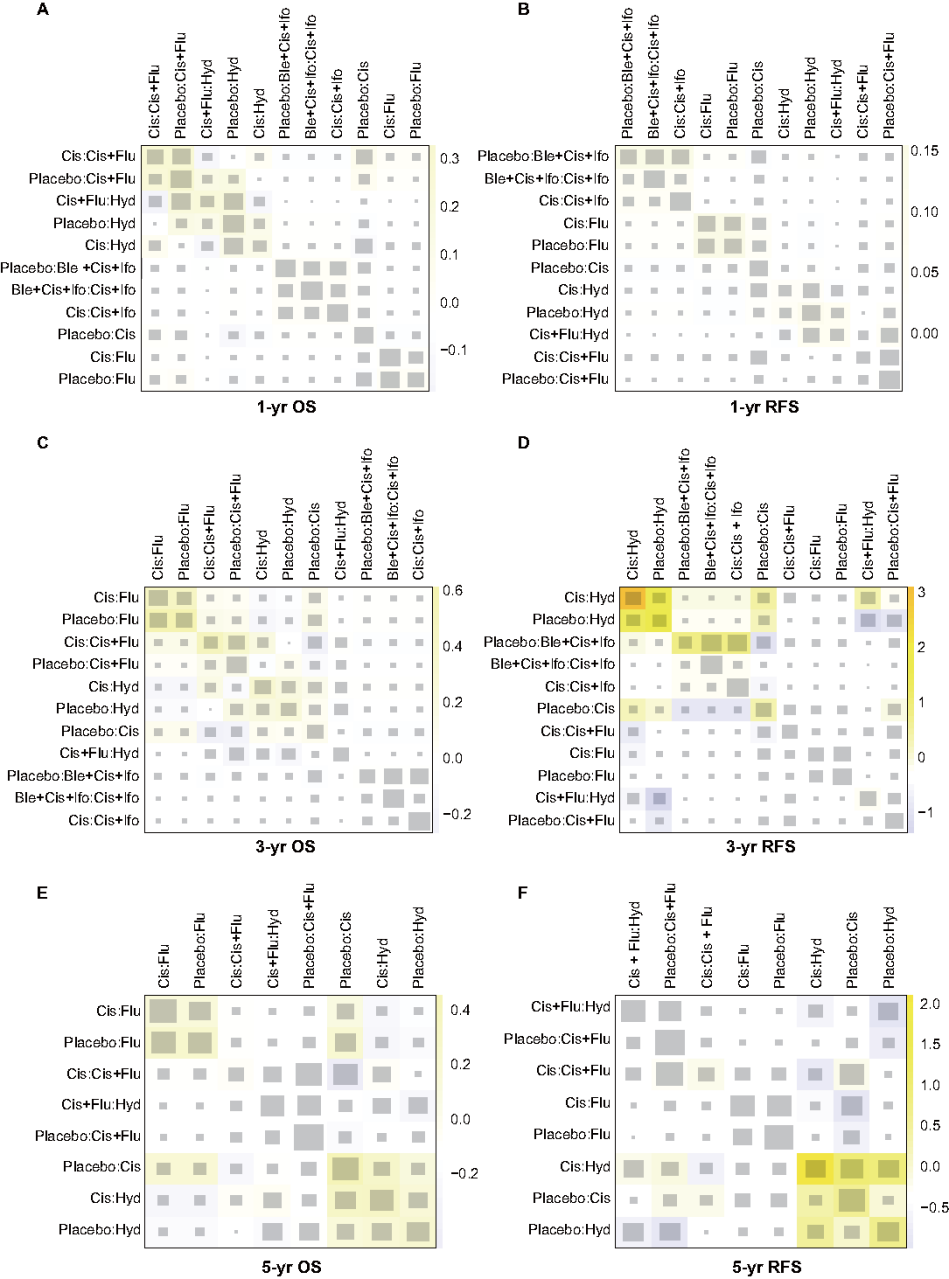
Netheat plots of inconsistency test for survival outcomes **(A)** 1-year overall survival (1-year OS); **(B)** 1-year recurrence-free survival (1year RFS); **(C)** 3-year OS; **(D)** 3-year RFS; **(E)** 5-year OS; **(F)** 5-year RFS.

**Figure 4:**
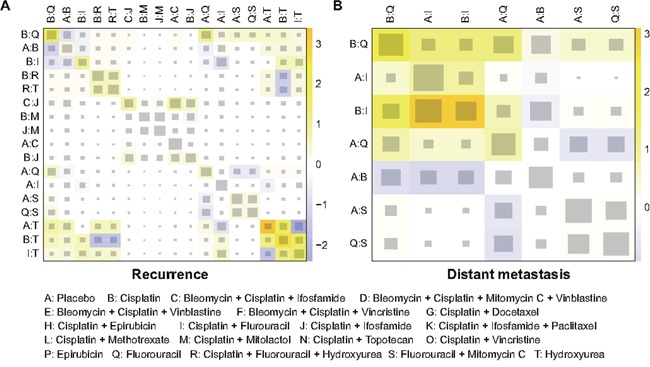
Netheat plots of inconsistency test for recurrence and distant metastasis **(A)** recurrence; **(B)** distant metastasis.

**Figure 5:**
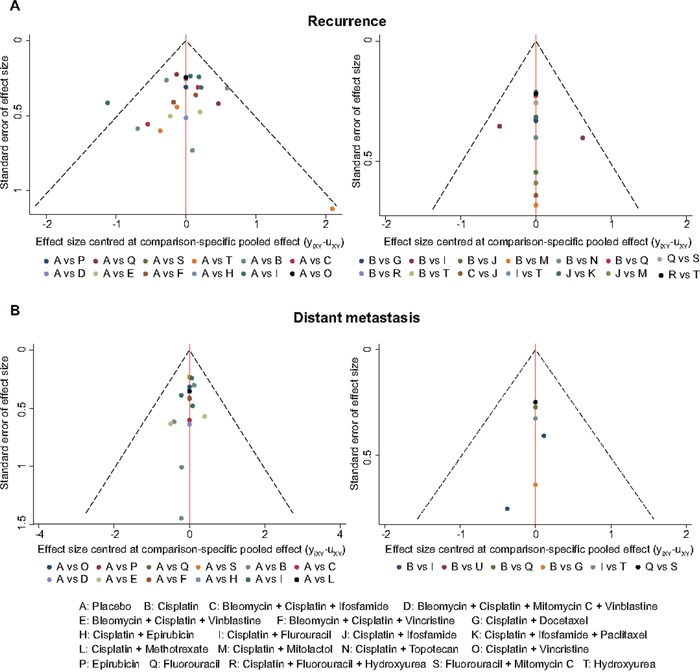
Comparison adjusted funnel plots of publication bias test for recurrence and distant metastasis **(A)** recurrence; **(B)** distant metastasis.

The heat plots in Figures 3–4 provided a detailed assessment of the inconsistency in this NMA. It appeared that there was no significant inconsistency identified within the net heat plot. Publication bias was visually using the comparison-adjusted funnel plots (Figure 5). We found no significant asymmetry patterns in funnel plots, so we concluded there were no significant publication bias in the included studies.

## DISCUSSION

To appraise the efficacy of usual chemotherapies when combined with radiotherapy as concurrent, adjuvant, or neoadjuvant treatment to patients with cervical cancer after surgery, 39 RCTs or clinical trials covering 22 interventions were analyzed, in terms of OS, RFS, the incidence of recurrence, and distant metastasis, in this NMA. It is the first issue in this domain, which integrated both the direct evidence and the indirect comparison remedying the insufficiency of the traditional meta-analysis.

In accordance with results, the combination of two or three medicines with diverse functional mechanisms had better impact than mono-chemotherapy. On the basis of biochemical mechanisms, relevant agents can be classified into four types, drugs affecting the structure and function of DNA, drugs interfering protein synthesis and function, drugs intercalating DNA to interfere the transcription as well as drugs used for metabolism inhibition. Statistically, drugs affecting the structure and function of DNA, including cisplatin, ifosfamide, bleomycin, and mitomycin C, were the most common medication used in cervix cancer treatment, but the specific mechanism of agents differed from each other, such as cisplatin can form a cross link between the guanines in the DNA after dissociation with chlorine [54, 55] while ifosfamide lead DNA during S phase to shape into cross link, which inhibits the growth and reproduction of tumor cells [56]. Antimetabolites, fluorouracil and hydroxyurea involved, are referred to as the drugs influencing biosynthesis of nucleic acid as well as being vital to the cell growth and proliferation [57, 58]. By distinct mechanisms, the ultimate purpose of both agents is impeding the synthesis of DNA [59–61]. With respect to the drugs acting on the necessary proteins as tubulin and ribosome, paclitaxel and docetaxel upset the dynamic equilibrium between tubulin and its dimer, accelerate the assembling of tubulin and interfere with its disassembly, therefore stop the cell cycle at G_2_/M phase [62–64]. Epirubicin can insert the base pairs of DNA, limiting the activity of transcriptase to influence the synthesis of mRNA, especially in the S and M phase of cell cycle [65]. Thus, the combination of multiple drugs with distinctive anti-cancer mechanisms would tend to exhibit superior efficacy especially to reduce the drug resistance during the long term administration.

This NMA assessed the efficacy of 22 chemotherapeutic treatments, containing nearly all clinical regular prescription in the aspects of 1-year, 3-year, and 5-year OS and RFS, as well as the incidence of recurrence and distant metastasis. However, there is still insufficiency that cannot be denied. First, the inclusion trials were limited, and 13 strategies were emerged only once, the inherent error of which were brought in this NMA. Next, the dose and delivery method of each agents were not considered to be an impact factor, but these factors did have influence on their efficacy. The discrepancy on 5-year RFS of the trials between Pearcey and Wong might be caused by the dosage of cisplatin [39]. Moreover, though the FIGO stages of subjects were listed, there was no discrimination in the course of data analysis. Nevertheless, in the clinical practice, the conditions of patients had an important impact on treatment results, including periaortic and pelvic lymph node status, patient age, performance status, bilateral disease and clinical stage [66]. For instance it is evident that the tumor size had the direct relation to the occurrence of metastasis and recurrence [67, 68]. Therefore, more authentic trials are needed and on the basis of colossal data, the particular subgroup analysis can be performed.

Overall, in consideration of the results of this NMA, cisplatin+fluorouracil+hydroxyurea, cisplatin+ docetaxel and fluorouracil+mitomycin C, were outstanding in prolonging the length of OS, whilemitomycin C, cisplatin+fluorouracil+hydroxyurea and cisplatin+docetaxel had good performance in RFS. Integrating both OS and RFS data, cisplatin+fluorouracil+hydroxyurea and cisplatin+docetaxel were highly recommended as first tier chemotherapies based on their equally preferable performance in long term. The first three with excellent performance in reducing the recurrence were cisplatin+ifosfamide, cisplatin+ifosfamide+paclitaxel, and cisplatin+docetaxel, in contrast to epirubicin which were beneficial to the significant decrease of distant metastasis. However, it should be noted that the individual conditions of patient should be taken into account thoroughly in clinical application.

## MATERIALS AND METHODS

### Search strategy

To obtain the relevant trial data, we searched electronic database PubMed, Embase and Cochrane Library for RCTs and clinical trials, regardless of the diversity of language, with the following key terms and their synonyms combined, “cervix cancer”, “radiotherapy”, “surgery”, and among “chemotherapy”, “concurrent chemotherapy”, “neoadjuvant chemotherapy”, “adjuvant chemotherapy”, and specific drugs, such as “cisplatin”, “fluorouracil”, “hydroxyurea” were included. Meanwhile, we also examined the reference lists of all the existed meta-analyses and systemic reviews, to guarantee the sample size of tested interventions. And all these work mentioned above were done by two reviewers individually.

### Inclusion and exclusion criteria

All the included trials must meet the listed criteria: (i) at least one of the involved interventions should be used to treat the cervix cancer of patients; (ii) the interventions could include concurrent chemotherapy, neoadjuvant chemotherapy, and adjuvant chemotherapy, but should be combined with radiotherapy and after surgery, while the strategy of radiotherapy and surgery were no limitations; (iii) the overall survival (OS) or the recurrence-free survival (RFS) should be compared between two interventions or interventions and placebo. Besides, although some trials satisfied the inclusion conditions, since they carried out between different methods of administration of the identical drug or the intervention they investigated which cannot form a loop, they were still excluded eventually. The trials included should also meet PRISMA guidelines. According to these criteria, two reviewers screened titles and abstracts of all retrieved articles, and the full texts would be examined respectively when necessary. And any arguments would be solved under discussion by the panel.

### Outcome measurements and data extraction

The basic features of this study, including author, year of publication, country, and the efficacy outcomes, were extracted from each eligible trial [69]. To assess the prognosis of chemotherapy aiming to cervix cancer, OS and RFS are the common outcomes. And quoting the interpretation from the NCI, OS indicates the length of time from the start of the chemotherapy for the cervix cancer, that patients diagnosed with the disease are still alive; and RFS means the length of time during and after the chemotherapy of the cervix cancer, that a patient lives without the diagnosed cancer. Except for the data given directly, the outcomes can also be extracted from the OS and RFS curves, the cumulative percentage versus time after chemotherapy administration. The incidence of recurrence and distant metastases were also evaluated as efficacy predictors.

### Statistical analysis

Based on the connection among treatments, four network plots on recurrence, distant metastasis, OS and RFS were drawn. The heterogeneity of fixed-effects model among each study effect was calculated through Cochran's Q and I squared statistic, which were presented in the net heat plots [70, 71]. Generally, if *P_h_* < 0.05 or *I*^2^ > 50%, it implied that a significant heterogeneity was existed, and then the fixed-effects model would be replaced by the random-effects model.

STATA 12.0 software and WinBUGS software were used for, which showed us the combination of direct and indirect evidence. In this NMA, we synthesized the direct and indirect evidence on 1-year, 3-year, 5-year OS and RFS, recurrence and distant metastasis. HR or OR with their 95% credible intervals (CrIs) were applied to evaluate the relative efficacy for specific comparison. To sort the chemotherapies according to their efficacies on recurrence and distant metastasis, the surface under the cumulative ranking curve (SUCRA) was applied, cumulating the percentages of each intervention with assuming the best, the second best and so on. And then, the optimal treatment would have the highest cumulative probability [72]. Furthermore, the heat plots for each outcome were performed to reflect the contribution of direct evidence to the network comparison and their inconsistency. Moreover, the publication bias on recurrence and distant metastasis were considered through the funnel plots, standard error of effect size versus the effect size centered at comparison-specific pooled effect.

## Abbreviations

HPV: human papillomavirus
RCTs: Randomized controlled trials
OS: overall survival
RFS: recurrence-free survival
HR: hazard ratio
OR: odds ratio
CI: confidence interval
SUCRA: surface under cumulative ranking curve
HPV: human papillomavirus
FIGO: Federation of Gynecology and Obstetrics
RCTs: randomized control trials
MA: meta-analyses
NMA: network meta-analysis
dUMP: deoxyuridine monophosphate
CrIs: credible intervals
Ble: Bleomycin
Cis: Cisplatin
Doc: Docetaxel
Epi: Epirubicin
Flu: Fluorouracil
Hyd: Hydroxyurea
Ifo: Ifosfamide
MitoC: Mitomycin C
Pac: Paclitaxel
Vbl: Vinblastine
Vcr: Vincristine
